# *Enterocytozoon bieneusi* genotypes in cats and dogs in Victoria, Australia

**DOI:** 10.1186/s12866-019-1563-y

**Published:** 2019-08-08

**Authors:** Yan Zhang, Anson V. Koehler, Tao Wang, David Cunliffe, Robin B. Gasser

**Affiliations:** 1 Department of Veterinary Biosciences, Melbourne Veterinary School, Faculty of Veterinary and Agricultural Sciences, The University of Melbourne, Parkville, Victoria 3010 Australia; 2 Lort Smith Animal Hospital, North Melbourne, Victoria 3051 Australia

**Keywords:** Cats, Dogs, *Enterocytozoon bieneusi*, Genotypes, Prevalence, Australia

## Abstract

**Background:**

*Enterocytozoon bieneusi* is one of the commonest microsporidians contributing to human microsporidiosis, and is frequently found in animals in various countries. However, there is limited epidemiological information on this microorganism in Australia. Here, we undertook the first molecular epidemiological study of *E. bieneusi* in cats and dogs in Victoria.

**Results:**

Genomic DNAs were extracted from 514 individual faecal deposits from cats (*n* = 172) and dogs (*n* = 342) and then tested using PCR-based sequencing of the internal transcribed spacer (*ITS*) of nuclear ribosomal DNA. Four distinct genotypes (designated D, PtEb IX, VIC_cat1 and VIC_dog1) of *E. bieneusi* were identified in 20 of the 514 faecal samples (3.9%). Genotype D is known to have a broad host range (humans and other animals) and has a wide geographical distribution around the world. The identification of this genotype here suggests that companion animals might represent reservoir hosts that are able to transmit *E. bieneusi* infection to humans in Australia. A phylogenetic analysis of *ITS* sequence data revealed that the novel genotype VIC_cat1 is related to the known genotype type IV within Group 1, and the new genotype VIC_dog1 is linked to a contentious “Group 3”, which includes genotypes reported previously in the published literature to represent Group 2 or 3.

**Conclusions:**

A future, large-scale phylogenetic study of all known *E. bieneusi* genotypes, including VIC_dog1, should aid in clarifying their relationships and assignment to Groups, and in the identification of new genotypes, thus assisting epidemiological investigations.

**Electronic supplementary material:**

The online version of this article (10.1186/s12866-019-1563-y) contains supplementary material, which is available to authorized users.

## Background

*Enterocytozoon bieneusi* (Microsporidia) was first detected in 1985 in a Haitian patient (HIV/AIDS) suffering from severe diarrhoea [1]. As an emerging infectious agent, *E. bieneusi* is the commonest species contributing to human microsporidiosis [2], characterised by symptoms of acute or chronic diarrhoea, malabsorption and/or wasting [3]. Spores of *E. bieneusi* infect epithelial cells, replicate (proliferative phase) and are then shed as new, mature spores (sporogonic phase) in faeces into the environment; these spores can contaminate water (e.g., drinking source water and wastewater) and food, posing a risk to public health [3, 4]. Therefore, *E. bieneusi* has been classified as a Category B Priority Pathogen by the United States Environmental Protection Agency (EPA) [5].

It is not possible to specifically identify microsporidians using microscopic methods [6], such that PCR-based sequencing of the internal transcribed spacer (*ITS*) of nuclear ribosomal DNA is employed to identify *E. bieneusi* to the species and genotypic levels [4]. Using molecular tools, *E. bieneusi* has been found in a broad host range, including humans and various orders of other animals (including mammals: Artiodactyla, Lagomorpha, Carnivora, Perissodactyla, Primates and Rodentia, and birds: Columbiformes, Falconiformes, Galliformes and Passeriformes) as well as in water and food samples [3]. Some genotypes of *E. bieneusi* (e.g., D and EbpC) commonly reported in humans [7] have also been found in animals [8, 9]. This aspect raises the questions as to whether animals with a close affiliation with people play a significant role in the transmission of *E. bieneusi* infection to humans.

Studies of *E. bieneusi* in humans and other animals have been conducted in ~ 40 countries [10], but until recently, there has been very little research on this microsporidian in Australia. In 2018, we investigated *E. bieneusi* in wild herbivores. First, we identified some potentially zoonotic genotypes of *E. bieneusi* in wild sambar deer (D, J, MWC_d1-d2 and Type IV) [10] and marsupials (MWC_m1 and NCF2) inhabiting Melbourne’s water catchments (MWCs) [11]. Then, we extended our studies to farmed animals, including cattle in one of the MWCs (i.e. Tarago) [12] as well as alpacas in six states of Australia [13], to explore the prevalence and zoonotic potential of *E. bieneusi* in these farmed animals. The results from these studies revealed five genotypes (BEB4, I, J and TAR_fc1 and TAR_fc2) from cattle and one (ALP1) from alpaca with potential to infect humans. More recently, a study of *E. bieneusi* in humans [14] showed that genotype ALP1, previously found exclusively in alpaca [13], was identified in humans in Australia, indicating that this camelid may act as a reservoir for transmission to people.

In the present study, we investigated the prevalence of *E. bieneusi* in companion animals (cats and dogs) with a relatively close association to humans in both urban and rural environments in the state of Victoria, Australia, and characterised genotypes and assessed their zoonotic potential using a molecular phylogenetic approach.

## Results

Using nested PCR-based sequencing of *ITS*, *E. bieneusi* DNA was detected in 20 of 514 (3.9%) faecal samples. Of these 20 test-positive samples, five (2.9% of 172) were from cats and 15 (4.4% of 342) from dogs (*P* = 0.478). Ten of them (7.9%; 10/126) were from juvenile cats (*n* = 4) and dogs (*n* = 6); and ten were from adults (2.7%; 10/371; including 1 cat and 9 dogs) (*P* = 0.016) (Tables 1 and 2). Of the 20 test-positive samples, nine (3.9%; 9/233) were from female cats (*n* = 2) and dogs (*n* = 7); and ten (3.7%; 10/270) were from male cats (n = 2) and dogs (*n* = 8), and one cat sample whose sex was not recorded (*P* = 1.000). Seven of these test-positive samples were collected in Spring and 13 in Summer (*P* = 0.494) (Table 2).

**Table 1:** Summary of information on faecal samples collected from household cats and dogs (age and sex) donated by an animal hospital located in Melbourne, Victoria, Australia, in Spring (September – November 2018) and Summer (December 2018 – February 2019)

Host Sex	Adult	Juvenile	Samples from animals of unknown age	Total prevalence of *E. bieneusi* in% (text-positive sample nos./total sample nos.)
Cat	111	55	6	2.9 (5/172)
Female	42	26	4	2.8 (2/72)
Male	65	24	2	2.2 (2/91)
NA	4	5	0	11.1 (1/9)
Dog	260	71	11	4.4 (15/342)
Female	125	31	5	4.3 (7/161)
Male	135	38	6	4.5 (8/179)
NA	0	2	0	0 (0/2)
Totals	371	126	17	3.9 (20/514)

*NA* not available

**Table 2:** The influence of the risk factors, including host species (domestic cat and dog), age (adult, juvenile), sex (female, male) and season (Spring, Summer), on *Enterocytozoon bieneusi* prevalence (by PCR-based sequencing of the internal transcribed spacer, *ITS*), assessed using the Chi-square and Fisher’s exact tests

Risk factors (host/age/sex/season)	No. of samples tested	No. of test-negative samples	No. of test-positive samples (%)	Odds ratio (95% CI)	*P-*value	Chi-square
Host species						
Cat	172	167	5 (2.9)	1.532 (0.547–4.288)	0.478	0.413
Dog	342	327	15 (4.4)			
Total	514	494	20 (3.9)			
Age group						
Adult	371	361	10 (2.7)	3.112 (1.264–7.663)	0.016*	0.010*
Juvenile	126	116	10 (7.9)			
Total	497	477	20 (4.0)			
Sex						
Female	233	224	9 (3.9)	0.957 (0.382–2.397)	1.000	0.926
Male	270	260	10 (3.7)			
Total	503	484	19 (3.8)			
Season						
Spring	227	220	7 (3.1)	1.491 (0.585–3.801)	0.494	0.400
Summer	287	274	13 (4.5)			
Total	514	494	20 (3.9)			

* = statistically significant (*P* < 0.05). *df* degree of freedom. Each df = 1 in this study

The strength of association was measured using an odds ratio calculated with 95% confidence intervals (95% CI), and statistical significance was given as a *P*-value

**Fig. 1:**
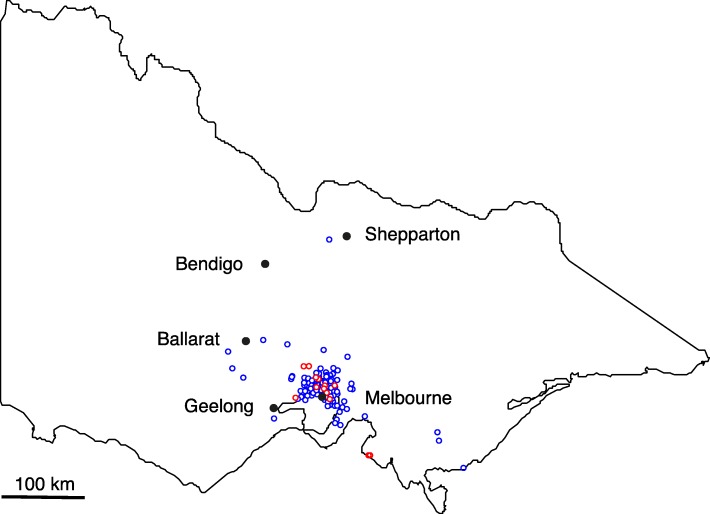
Map indicating the geographical origins of individual cats and dogs from which faecal samples were collected in Victoria, Australia. Closed circles = locations from where faecal samples were collected. Blue open circles = *Enterocytozoon bieneusi* test-negative samples. Red open circles = *E. bieneusi* test-positive samples. Black solid circles = major cities in Victoria

Most test-positive samples were from North Melbourne (*n* = 6), followed by Gladstone Park (~ 14 km from Lort Smith Animal Hospital), Toorak (8 km), and Woolamai (*n* = 2 each) (83 km), Collingwood (3 km), Glenroy (11 km), Northcote (5 km), Sunbury (36 km), Templestowe (17 km), Wildwood (27 km), Wyndham Vale (30 km), Yarraville (*n* = 1 each) (6 km), of which only Gladstone Park (n = 2), Sunbury (n = 1), Wildwood (n = 1), Woolamai (n = 2) and Wyndham Vale (n = 1) are not in the city of Melbourne (Fig. 1). There was no significant association of *E. bieneusi* prevalence with host species (*P* = 0.478), sex (*P* = 1.000) and season (*P* = 0.494). However, there was a significant association between age and prevalence for *E. bieneusi* (*P* = 0.016), with juvenile animals having 3.11-times higher risk of *E. bieneusi*-positivity than adults (OR = 3.11; 95% CI [1.264–7.663]) (Table 2).

The sequencing of the 20 individual *ITS* amplicons (241–244 bp) and their subsequent comparisons with reference sequences from the GenBank database revealed two known genotypes of *E. bieneusi* (D and PtEb IX) and two novel genotypes (called VIC_cat1 and VIC_dog1) (Table 3). The sequences determined unequivocally represented genotypes PtEb IX (50%; 10/20), D (40%; 8/20), followed by VIC_cat1 (5%; 1/20) and VIC_dog1 (5%; 1/20).

**Table 3:** Genotypes of *Enterocytozoon bieneusi* characterised by nested PCR-based sequencing of the internal transcribed spacer (*ITS*) region of nuclear ribosomal DNA from 514 domestic cats and dogs (different ages and sexes) in Victoria, Australia (Sep. 2018 - Feb. 2019)

Genotypic designation	GenBank accession no.	Sample code	Host	Location	Age	Sex
D	MK696083	LS0011	Dog	North Melbourne	J	F
D	a	LS0008	Cat	Toorak	J	NA
D	a	LS0019	Cat	Toorak	J	M
D	a	LS0055	Cat	North Melbourne	J	F
D	a	LS0174	Dog	Woolamai	J	M
D	a	LS0189	Dog	Woolamai	J	F
D	a	LS0227	Dog	North Melbourne	A	M
D	a	LS0317	Dog	Glenroy	A	F
PtEb IX	MK696084	LS0083	Dog	Wildwood	J	M
PtEb IX	b	LS0228	Dog	Northcote	A	F
PtEb IX	b	LS0232	Dog	Wyndham Vale	A	M
PtEb IX	b	LS0249	Cat	Yarraville	J	M
PtEb IX	b	LS0278	Dog	Collingwood	A	M
PtEb IX	b	LS0291	Dog	Gladstone Park	J	M
PtEb IX	b	LS0318	Dog	Gladstone Park	A	F
PtEb IX	b	LS0337	Dog	North Melbourne	A	F
PtEb IX	b	LS0355	Dog	North Melbourne	A	M
PtEb IX	b	LS0439	Dog	Sunbury	A	F
VIC_cat1*	MK696086	LS0421	Cat	Templestowe	A	F
VIC_dog1*	MK696085	LS0336	Dog	North Melbourne	J	M

* novel genotype. *A* adult, *F* female, *J* juvenile, *M* male, *NA* not available

^a^ sequence identical to that of MK696083. ^b^ sequence identical to that of MK696084

The *ITS* sequences from amplicons representing genotypes D (synonyms: CEbC, MJ10–12, NCF7, Peru9, PigEBITS9, PtEb VI, SHW1 and WL8) and genotype PtEb IX (synonyms: eb52 and EntcanA) were identical to those with accession nos. AF101200 (derived from human) (*ITS*; 243 bp) and DQ885585 (dog) (*ITS*; 244 bp), respectively (Additional file 2: Table S2). The *ITS* sequences from two amplicons representing VIC_cat1 (accession no. MK696086) and VIC_dog1 (accession no. MK696085) were one to two nucleotides different from the sequences with accession nos. AF242478 (type IV; human) and HM992519 (genotype CHN10; pig), respectively.

**Fig. 2:**
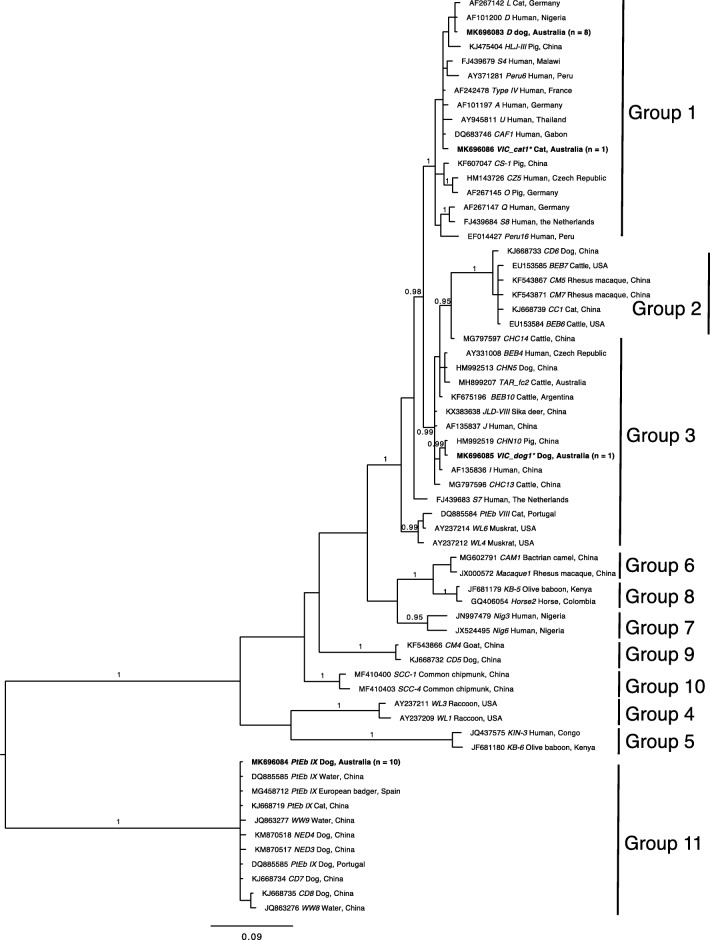
Phylogenetic analysis of internal transcribed spacer (*ITS*) of nuclear ribosomal DNA sequence data (cf. Additional file 1: Table S1) by Bayesian inference (BI). Included here are *ITS* sequences of (i) *E. bieneusi* genotypes representing all currently recognised Groups (1 to 10) from the published literature, (ii) four genotypes of *Enterocytozoon* identified in the present study (bold-type) and (iii) the outgroup taxa. Statistically significant posterior probabilities (pp) of > 0.95 are indicated on branches. The scale-bar represents the number of substitutions per site. * = novel genotype

The *ITS* sequences of all four genotypes defined herein were aligned with sequences representing all ten established Groups of *E. bieneusi* [9, 15, 16], and then subjected to phylogenetic analysis (Fig. 2). In this analysis, Groups 1 to 10 were each strongly supported (pp = 0.95 to 1.00). Based on this analysis, genotype D and VIC_cat1 were assigned to Group 1 (pp = 1.00); genotype VIC_dog1 clustered with Group 3 (pp = 0.99); and genotype PtEb IX fell into Group 11 (outgroup) [14] with strong statistical support (pp = 1.00). Traditionally, *E. bieneusi* genotypes from dog, namely PtEb IX (accession no. DQ885585), CD7 (KJ668734) and CD8 (KJ668735), were commonly used as outgroup taxa in phylogenetic analyses, as their *ITS* sequences are quite distinct from *E. bieneusi* representing Groups 1–10.

## Discussion

This is the first molecular investigation of *E. bieneusi* from companion animals in Australia. Here, we established the genetic identity of *E. bieneusi* in faecal DNA samples from domestic cats and dogs using nested PCR-based sequencing of the *ITS* region. Globally, the prevalence of *E. bieneusi* in cats ranges from 1.4% (2/143) (household and stray) [17] to 31.3% (25/80) [18], while that in household and stray dogs ranges from 2.5% (2/79) [19] to 15.5% (54/348) [20] (Additional file 3: Table S3). By contrast, the prevalence of *E. bieneusi* in cats (2.9%) and dogs (4.4%) in our study was relatively low. The present study was designed to eliminate the possibility of genetically characterising *E. bieneusi* relating to endemic infections within an animal hospital environment - all faecal samples were collected from newly admitted animals. Therefore, the low prevalence of *E. bieneusi* in this study is plausible, as in other studies faecal samples were collected from pet markets or farms, where cats or dogs were raised together [16, 21], or from stray cats or dogs living under poor conditions [22]. Also important to consider is that various environmental factors (e.g., temperature and UV radiation), immunity, host age and sex, sample size and location may influence the prevalence rate.

Association analysis revealed that juvenile cats and dogs were significantly associated with a higher percentage of *E. bieneusi* prevalence than adults. This finding is supported by a previous study by Phrompraphai et al. [23], who conducted association analyses between *E. bieneusi* prevalence in dogs and their age, with dogs of less than one year of age having a higher prevalence of *E. bieneusi* infection. Some studies of *E. bieneusi* in other animals (e.g., cattle, deer, sheep and pig) [9, 24–27] and humans [14] have also revealed that age is a risk factor that might influence the prevalence of *E. bieneusi* infection. Host-age inversely associates with *E. bieneusi* prevalence, which could relate to immature immune systems in young animals. An association between *E. bieneusi* prevalence and geographical region could not be established due to an inadequate number of test-positive samples in individual localities.

The analysis of the *ITS* sequences derived from 20 faecal samples revealed four genotypes, including two known genotypes - D and PtEb IX (from both cats and dogs) - and two novel genotypes - VIC_cat1 and VIC_dog1. This is the first time that genotype PtEb IX, one of the most divergent genotypes of *E. bieneusi*, has been identified in Australia. An appraisal of current literature shows that PtEb IX appears to have a relatively narrow host-range, including dogs, cats and the European badger [28], with this genotype being principally found in dogs (Additional file 2: Table S2). This information suggests that genotype PtEb IX is transmitted predominantly among dogs, and is not likely to play a role in *E. bieneusi* transmission to humans. By contrast, genotype D is one of the commonest genotypes, and has a very broad host-range and a world-wide distribution [4]. This genotype has been found in more than 50 species of animals (Additional file 2: Table S2), including humans, as well as in water and foods, indicating that genotype D can be transmitted between humans and other animals (and vice versa), probably involving direct contact and/or the ingestion of *E. bieneusi-*contaminated water or food.

The identification of genotype D in cats and dogs in this study demonstrates that these companion animals likely serve as host reservoirs for selected *E. bieneusi* genotypes which are potentially transmissible to humans, particularly pet owners, due to close contact with their animals. Apart from genotype D, genotypes ALP3, Hum_q1–q3 and Ind4 (in the States of Queensland and Western Australia) [14] and genotype B (in the State of New South Wales) have been found in Australian people [2]. However, no studies of humans have yet been conducted in Victoria. Therefore, future studies of *E. bieneusi* in humans, especially those with and without pet cats and dogs, are warranted to establish whether “zoonotic” genotypes of *E. bieneusi* found in humans match those of their cats or dogs.

Phylogenetic analysis of the present *ITS* sequence data and selected sequences representing the ten established Groups of *E. bieneusi* (Fig. 2) revealed that novel genotype VIC_cat1 was most closely related to type IV, which has been frequently detected in humans and animals and represents Group 1, suggesting that genotype VIC_cat1 has zoonotic potential. However, the other novel genotype, VIC_dog1, that clustered with genotype CHN10, falls within a contentious “Group 3”, which includes genotypes reported previously to represent Group 2 or 3 [15, 16]. For instance, genotypes BEB4 and CHN10 have been recorded in Group 2, and genotypes S7 and WL4 have been placed in Group 3 (Additional file 1: Table S1). The ambiguous position of this novel genotype might lead to imprecise interpretations or conclusions regarding its epidemiological significance. Therefore, in our opinion, it would be useful to conduct a large-scale study of all unique *ITS* sequences (~ 600) reported and published to date, to search for patterns of nucleotide alterations in an alignment of all of these sequences, and to carry out a comprehensive phylogenetic analysis to untangle contradictions or confusions in the relationships of genotypes and Groups. Such an analysis would provide a refined framework for the assignment of new genotypes to Groups and would assist in assessing zoonotic potential in relation to members within Groups 2 to 10.

## Conclusions

Exploring the genetic composition of *E. bieneusi* populations in animals and humans is important for understanding transmission patterns of disease (microsporidiosis), and for the prevention and control of this disease. By conducting the present molecular-phylogenetic investigation of *E. bieneusi ITS* rDNA sequences derived from faecal samples (*n* = 514) from household cats and dogs in Australia, we identified two known genotypes (D and PtEb IX) and two new genotypes VIC_cat1 and VIC_dog1. Genotypes D and VIC_cat1 both have zoonotic potential, suggesting that companion animals carrying these genotypes could be reservoirs for infection to humans*.* The novel genotype VIC_dog1 falls within a contentious Group, prompting the need for a future, large-scale molecular-phylogenetic analysis of all currently known *E. bieneusi* genotypes.

## Methods

A total of 514 faecal samples from household cats (*Felis catus*; *n* = 172) and dogs (*Canis lupus familiaris*; *n* = 342), undergoing medical treatment, were donated by the Lort Smith Animal Hospital in North Melbourne between September 2018 and February 2019 (Table 1). In this hospital, fresh faecal samples were taken from newly admitted animals. Most of the animals (94.2%) were from the urban and suburban areas in Melbourne and environs, while others (5.1%) were from rural or regional towns in the State of Victoria (Fig. 1). Samples were from adult cats (*n* = 111) and dogs (*n* = 160); juvenile cats (*n* = 55) and dogs (*n* = 71); and from animals of unknown age (n = 17). Samples represented female cats (*n* = 72) and dogs (*n* = 161); male cats (*n* = 91) and dogs (*n* = 179); and a small number (n = 11) came from animals whose sex was not recorded (Table 1).

Genomic DNAs were extracted directly from 0.25 g of individual faecal samples using the PowerSoil kit (MoBio, USA), according to the manufacturer’s instructions. The *ITS* region of *E. bieneusi* was amplified from individual genomic DNAs by nested PCR using the primers MSP-1 (forward: 5′-TGA ATG KGT CCC TGT-3′) and MSP-2B (reverse: 5′-GTT CAT TCG CAC TAC T-3′) in the first round, and using primers MSP-3 (forward: 5′-GGA ATT CAC ACC GCC CGT CRY TAT-3′) and MSP-4B (reverse: 5′-CCA AGC TTA TGC TTA AGT CCA GGG AG-3′) in the second round [10]. Essential positive and negative controls were included in each PCR run.

Following column-purification, PCR products were directly sequenced [10]. The *ITS* sequences obtained in this study (publicly available under GenBank accession nos. MK696083–MK696086) were inspected for quality and compared with reference sequences acquired from the GenBank database (Additional file 1: Table S1). Genotypes of *E. bieneusi* were named in accordance with recommendations made by Santín and Fayer [4, 29].

All *ITS* sequences obtained from the present study, together with reference sequences were aligned, and subjected to phylogenetic analysis using using the Bayesian inference (BI) and Monte Carlo Markov Chain (MCMC) methods in MrBayes v.3.2.3 [10]. Posterior probability (pp) values were calculated by running 2,000,000 generations with four simultaneous tree-building chains, with trees being saved every one hundredth generation. A 50% majority rule consensus tree for each analysis was constructed based on the final 75% of trees generated by BI [10]. Genotypes were classified into groups using a recognised classification system [15, 16]. Chi-square and Fisher’s exact tests were utilised to test the association between *E. bieneusi* prevalence and possible risk factors (age, host, season and sex). The odds ratio (OR), calculated with a 95% confidence interval (95% CI), was used to measure the strength of association between the prevalence of *E. bieneusi* and a univariate risk factor. A *P*-value of < 0.05 was considered statistically significant. The SPSS Statistics package 25.0 (IBM, SPSS Inc., Chicago, IL) was used for all statistical analyses [14].

## Additional files

Additional file 1: **Table S1.** GenBank accession numbers of all internal transcribed spacer (*ITS*) of nuclear ribosomal DNA sequences used for phylogenetic analysis (Fig. 2), and associated information. Included here are *ITS* sequences of (i) *E. bieneusi* genotypes representing currently recognised Groups (1 to 10) from the published literature and genotypes without group assignment; (ii) four genotypes of *Enterocytozoon* identified/defined in the present study; and (iii) seven genotypes from the outgroups. (DOCX 94 kb)

Additional file 2: **Table S2.** Genotypes D (synonyms: CEbC, MJ10-12, NCF7, Peru9, PigEBITS9, PtEb VI, SHW1and WL8) and genotype PtEb IX (synonyms: eb52 and EntcanA) of *Enterocytozoon bieneusi* recorded in different host species, food and water samples fromprevious publications. (DOCX 284 kb)

Additional file 3: **Table S3.** All *Enterocytozoon bieneusi* genotypes, prevalence and risk factors recorded previously in cats (*Felis catus*) and dogs (*Canis lupus familiaris*) worldwide. (DOCX 75 kb)

## Data Availability

Nucleotide sequences reported in this paper are available in the GenBank database under accession numbers MK696083–MK696086.

## Abbreviations

CI: Confidence interval
*E. bieneusi*: *Enterocytozoon bieneusi*
*ITS*: Internal transcribed spacer of nuclear ribosomal DNA
MWC: Melbourne water catchments
pp: Posterior probability
