# Supplementary material for: Enterocytozoon bieneusi genotypes in cats and dogs in Victoria, Australia
Source: BMC Microbiol. 2019 Aug 8;19:183. doi: 10.1186/s12866-019-1563-y (PMC6686557; doi:10.1186/s12866-019-1563-y)
Supplement: Supplementary file 1 — Table S1. GenBank accession numbers of all internal transcribed spacer (ITS) of nuclear ribosomal DNA sequences used for phylogenetic analysis (Fig. 2), and associated information. Included here are ITS sequences of (i) E. bieneusi genotypes representing currently recognised Groups (1 to 10) from the published literature and genotypes without group assignment; (ii) four genotypes of Enterocytozoon identified/defined in the present study; and (iii) seven genotypes from the outgroups. (DOCX 94 kb) [file 12866_2019_1563_MOESM1_ESM.docx]

**Additional file 1: Table S1.** GenBank accession numbers of all internal transcribed spacer (*ITS*) of nuclear ribosomal DNA sequences used for phylogenetic analysis (Fig. 2), and associated information. Included here are *ITS* sequences of (i) *E. bieneusi* genotypes representing currently recognised Groups (1 to 10) from the published literature and genotypes without group assignment; (ii) four genotypes of *Enterocytozoon* identified/defined in the present study; and (iii) seven genotypes from the outgroups

| GenBank ID | Genotype | Origin | Country | Gro-up | Refer-ence |
| --- | --- | --- | --- | --- | --- |
| AF101200 | D | *Homo sapiens* (human) | Nigeria | 1a | [1] |
| AF267142 | L | *Felis catus* (cat) | Germany | 1a | [2] |
| AY371281 | Peru6 | *Homo sapiens* (human) | Peru | 1b | [3] |
| FJ439679 | S4 | *Homo sapiens* (human) | Malawi | 1b | [4] |
| AF101197 | A | *Homo sapiens* (human) | Germany | 1c | [5] |
| AF242478 | Type IV | *Homo sapiens* (human) | France | 1c | [6] |
| KF607047 | CS-1 | *Sus scrofa domesticus* (pig) | China | 1d | [7] |
| AY945811 | U | *Homo sapiens* (human) | Thailand | 1d | [8] |
| HM143726 | CZ5 | *Homo sapiens* (human) | Czech Republic | 1e | [9] |
| AF267145 | O | *Homo sapiens* (human) | Thailand | 1e | [2] |
| AF267147 | Q | *Homo sapiens* (human) | Germany | 1f | [2] |
| FJ439684 | S8 | *Homo sapiens* (human) | The Netherlands | 1f | [4] |
| DQ683746 | CAF1 | *Homo sapiens* (human) | Gabon | 1g | [10] |
| KJ475404 | HLJ-III | *Sus scrofa domesticus* (pig) | China | 1g | [11] |
| EF014427 | Peru16 | *Homo sapiens* (human) | Peru | 1h | [12] |
| EU153585 | BEB7 | *Bos taurus* (cattle) | USA | 2 | [13] |
| KF543867 | CM5 | *Macaca mulatta*  (rhesus macaque) | China | 2 | [14] |
| KF543871 | CM7 | *Macaca mulatta*  (rhesus macaque) | China | 2 | [14] |
| KJ668739 | CC1 | *Felis catus* (cat) | China | 2 | [15] |
| KJ668733 | CD6 | *Canis lupus familiaris* (dog) | China | 2 | [15] |
| EU153584 | BEB6 | *Cervus elaphus* (red deer) | China | 2 | [16] |
| AY331008 | BEB4 | *Homo sapiens* (human) | Czech Republic | 3 | [17] |
| KF675196 | BEB10 | *Bos taurus* (cattle) | Argenitina | 3 | [18] |
| MG797596 | CHC13 | *Bos taurus* (cattle) | China | 3 | [19] |
| MG797597 | CHC14 | *Bos taurus* (cattle) | China | 3 | [19] |
| HM992513 | CHN5 | *Canis lupus familiaris* (dog) | China | 3 | [20] |
| HM992519 | CHN10 | *Sus scrofa domesticus* (pig) | China | 3 | [20] |
| AF135836 | I | *Homo sapiens* (human) | China | 3 | [20] |
| AF135837 | J | *Homo sapiens* (human) | China | 3 | [20] |
| KX383638 | JLD-VIII | *Cervus nippon* (sika deer) | China | 3 | [21] |
| DQ885584 | PtEb VIII | *Felis catus* (cat) | Portugal | 3 | [21] |
| FJ439683 | S7 | *Homo sapiens* (human) | The Netherlands | 3 | [4] |
| MH899207 | TAR_fc2 | *Bos taurus* (cattle) | Australia | 3 | [22] |
| AY237212 | WL4 | *Ondatra zibethicus* (muskrat) | USA | 3 | [23] |
| AY237214 | WL6 | *Ondatra zibethicus* (muskrat) | USA | 3 | [23] |
| AY237209 | WL1 | *Procyon lotor* (raccoon) | USA | 4 | [23] |
| AY237211 | WL3 | *Procyon lotor* (raccoon) | USA | 4 | [23] |
| JF681180 | KB-6 | *Papio Anubis* (olive baboon) | Kenya | 5 | [24] |
| JQ437575 | KIN-3 | *Homo sapiens* (human) | Congo | 5 | [25] |
| JX000572 | Macaque1 | *Macaca mulatta*  (Rhesus macaque) | China | 6 | [26] |
| MG602791 | CAM1 | *Camelus bactrianus* (Bactrian camel) | China | 6 | [27] |
| JN997479 | Nig3 | *Homo sapiens* (human) | Nigeria | 7 | [28] |
| JX524495 | Nig6 | *Homo sapiens* (human) | Nigeria | 7 | [1] |
| GQ406054 | Horse 2 | *Equus ferus caballus* (horse) Colombia | | 8 | [29] |
| JF681179 | KB-5 | *Papio anubis* (olive baboon) Kenya | | 8 | [24] |
| KJ668732 | CD5 | *Canis lupus familiaris* (dog) | China | 9 | [15] |
| KF543866 | CM4 | *Capra aegagrus hircus* (goat) China | | 9 | [30] |
| MF410400 | SCC-1 | *Eutamias asiaticus*  (common chipmunk) | China | 10 | [31] |
| MF410403 | SCC-4 | *Eutamias asiaticus*  (common chipmunk) | China | 10 | [31] |
| MK696083 | D | *Canis lupus familiaris* (dog) | Australia | 1 | TS |
| MK696084 | PtEb IX | *Canis lupus familiaris* (dog) | Australia | OG | TS |
| MK696086 | VIC_cat1 | *Felis catus* (cat) | Australia | 1 | TS |
| MK696085 | VIC_dog1 | *Canis lupus familiaris* (dog) |  | 3 | TS |
| KJ668734 | CD7 | *Canis lupus familiaris* (dog) | China | OG | [15] |
| KJ668735 | CD8 | *Canis lupus familiaris* (dog) | China | OG | [15] |
| KM870517 | NED3 | *Canis lupus familiaris* (dog) | China | OG | [32] |
| KM870518 | NED4 | *Canis lupus familiaris* (dog) | China | OG | [32] |
| KJ668719 | PtEb IX | *Felis catus* (cat) | China | OG | [15] |
| MG458712 | PtEb IX | *Meles meles*  (European badger) | Spain | OG | [33] |
| DQ885585 | PtEb IX | Water | China | OG | [34] |
| DQ885585 | PtEb IX | *Canis lupus familiaris* (dog) | USA | OG | [35] |
| JQ863276 | WW8 | *Canis lupus familiaris* (dog) | Switzerland | OG | [36] |
| JQ863277 | WW9 | *Felis catus* (cat) | Poland | OG | [37] |

* = a novel genotype identified in the present study. NA = not available. TS = this study. OG = outgroup.

**References**

1. Akinbo FO, Okaka CE, Omoregie R, Adamu H, Xiao L. Unusual *Enterocytozoon bieneusi* genotypes and *Cryptosporidium hominis* subtypes in HIV-infected patients on highly active antiretroviral therapy. Am J Trop Med Hyg. 2013;89(1):157-61.

2. Dengjel B, Zahler M, Hermanns W, Heinritzi K, Spillmann T, Thomschke A, Loscher T, Gothe R, Rinder H. Zoonotic potential of *Enterocytozoon bieneusi*. J Clin Microbiol. 2001;39(12):4495-9.

3. Sulaiman IM, Bern C, Gilman R, Cama V, Kawai V, Vargas D, Ticona E, Vivar A, Xiao L. A molecular biologic study of *Enterocytozoon bieneusi* in HIV-infected patients in Lima, Peru. J Eukaryot Microbiol. 2003;50 Suppl 1: 591-6.

4. ten Hove RJ, Van Lieshout L, Beadsworth MB, Perez MA, Spee K, Claas EC, Verweij JJ. Characterization of genotypes of *Enterocytozoon bieneusi* in immunosuppressed and immunocompetent patient groups. J Eukaryot Microbiol. 2009;56(4):388-93.

5. Rinder H, Katzwinkel-Wladarsch S, Loscher T. Evidence for the existence of genetically distinct strains of *Enterocytozoon bieneusi*. Parasitol Res. 1997;83(7):670-2.

6. Liguory O, Sarfati C, Derouin F, Molina JM. Evidence of different *Enterocytozoon bieneusi* genotypes in patients with and without human immunodeficiency virus infection. J Clin Microbiol. 2001;39(7):2672-4.

7. Li W, Diao R, Yang J, Xiao L, Lu Y, Li Y, Song M. High diversity of human-pathogenic *Enterocytozoon bieneusi* genotypes in swine in northeast China. Parasitol Res. 2014;113(3):1147-53.

8. Leelayoova S, Subrungruang I, Suputtamongkol Y, Worapong J, Petmitr PC, Mungthin M. Identification of genotypes of *Enterocytozoon bieneusi* from stool samples from human immunodeficiency virus-infected patients in Thailand. J Clin Microbiol. 2006;44(8):3001-4.

9. Sak B, Kváč M, Kučerová Z, Květoňová D, Saková K. Latent microsporidial infection in immunocompetent individuals - a longitudinal study. PLoS Negl Trop Dis. 2011;5(5) :e1162.

10. Breton J, Bart-Delabesse E, Biligui S, Carbone A, Seiller X, Okome-Nkoumou M, Nzamba C, Kombila M, Accoceberry I, Thellier M. New highly divergent rRNA sequence among biodiverse genotypes of *Enterocytozoon bieneusi* strains isolated from humans in Gabon and Cameroon. J Clin Microbiol. 2007;45(8):2580-9.

11. Zhao W, Zhang W, Yang F, Cao J, Liu H, Yang D, Shen Y, Liu A. High prevalence of *Enterocytozoon bieneusi* in asymptomatic pigs and assessment of zoonotic risk at the genotype level. Appl Environ Microbiol. 2014;80(12):3699-707.

12. Cama VA, Pearson J, Cabrera L, Pacheco L, Gilman R, Meyer S, Ortega Y, Xiao L. Transmission of *Enterocytozoon bieneusi* between a child and guinea pigs. J Clin Microbiol. 2007;45(8):2708-10.

13. Fayer R, Santín M, Trout JM. *Enterocytozoon bieneusi* in mature dairy cattle on farms in the eastern United States. Parasitol Res. 2007;102(1):15-20.

14. Karim MR, Wang R, Dong H, Zhang L, Li J, Zhang S, Rume FI, Qi M, Jian F, Sun M, et al. Genetic polymorphism and zoonotic potential of *Enterocytozoon bieneusi* fom nonhuman primates in China. Appl Environ Microbiol. 2014;80(6):1893-8.

15. Karim MR, Dong H, Yu F, Jian F, Zhang L, Wang R, Zhang S, Rume FI, Ning C, Xiao L. Genetic diversity in *Enterocytozoon bieneusi* isolates from dogs and cats in China: host specificity and public health implications. J Clin Microbiol. 2014;52(9):3297-302.

16. Zhao W, Zhang W, Wang R, Liu W, Liu A, Yang D, Yang F, Karim MR, Zhang L. *Enterocytozoon bieneusi* in sika deer (*Cervus nippon*) and red deer (*Cervus elaphus*): deer specificity and zoonotic potential of ITS genotypes. Parasitol Res. 2014;113(11):4243-50.

17. Sak B, Brady D, Pelikánová M, Květoňová D, Rost M, Kostka M, Tolarová V, Hůzová Z, Kváč M. Unapparent microsporidial infection among immunocompetent humans in the Czech Republic. J Clin Microbiol. 2011;49(3):1064-70.

18. Del Coco VF, Córdobaa MA, Bilbao G, de Almeida Castro P, Basualdo JA, Santín M. First report of *Enterocytozoon bieneusi* from dairy cattle in Argentina. Vet Parasitol. 2014;199(1):112-5.

19. Yu F, Qi M, Zhao Z, Lv C, Wang Y, Wang R, Zhang L. The potential role of synanthropic rodents and flies in the transmission of *Enterocytozoon bieneusi* on a dairy cattle farm in China. J Eukaryot Microbiol. 2018; <https://doi.org/10.1111/jeu.12687>. Accessed 6 September 2018.

20. Zhang X, Wang Z, Su Y, Liang X, Sun X, Peng S, Lu H, Jiang N, Yin J, Xiang M, et al. Identification and genotyping of *Enterocytozoon bieneusi* in China. J Clin Microbiol. 2011;49(5):2006-8.

21. Lobo ML, Xiao L, Cama V, Stevens T, Antunes F, Matos O. Genotypes of *Enterocytozoon bieneusi* in mammals in Portugal. J Eukaryot Microbiol. 2006;53 Suppl 1: 61-4.

22. Zhang Y, Koehler AV, Wang T, Haydon SR, Gasser RB. *Enterocytozoon bieneusi* genotypes in cattle on farms located within a water catchment area. J Eukaryot Microbiol. 2018; https://doi.org/10.1111/jeu.12696. Accessed 25 October 2018.

23. Sulaiman IM, Fayer R, Lal AA, Trout JM, Schaefer FW, Xiao L. Molecular characterization of microsporidia indicates that wild mammals harbor host-adapted *Enterocytozoon* spp. as well as human-pathogenic *Enterocytozoon bieneusi*. Appl Environ Microbiol. 2003;69(8):4495-501.

24. Li W, Kiulia NM, Mwenda JM, Nyachieo A, Taylor MB, Zhang X, Xiao L. *Cyclospora papionis*, *Cryptosporidium hominis*, and human-pathogenic *Enterocytozoon bieneusi* in captive baboons in Kenya. J Clin Microbiol. 2011;49(12):4326-9.

25. Wumba R, Longo-Mbenza B, Menotti J, Mandina M, Kintoki F, Situakibanza NH, Kakicha MK, Zanga J, Mbanzulu-Makola K, Nseka T, et al. Epidemiology, clinical, immune, and molecular profiles of microsporidiosis and cryptosporidiosis among HIV/AIDS patients. Int J Gen Med. 2012;5:603-11.

26. Ye J, Xiao L, Ma J, Guo M, Liu L, Feng Y. Anthroponotic enteric parasites in monkeys in public park, China. Emerg Infect Dis. 2012;18(10):1640-3.

27. Qi M, Li J, Zhao A, Cui Z, Wei Z, Jing B, Zhang L. Host specificity of *Enterocytozoon bieneusi* genotypes in bactrian camels (*Camelus bactrianus*) in China. Parasit Vectors. 2018;11(1):219.

28. Akinbo FO, Okaka CE, Omoregie R, Dearen T, Leon ET, Xiao L. Molecular epidemiologic characterization of *Enterocytozoon bieneusi* in HIV-infected persons in Benin city, Nigeria. Am J Trop Med Hyg. 2012;86(3):441-5.

29. Santín M, Cortés Vecino JA, Fayer R. A zoonotic genotype of *Enterocytozoon bieneusi* in horses. J Parasitol. 2010;96(1):157-61.

30. Shi K, Li M, Wang X, Li J, Karim MR, Wang R, Zhang L, Jian F, Ning C. Molecular survey of *Enterocytozoon bieneusi* in sheep and goats in China. Parasit Vectors. 2016;9(1):23.

31. Deng L, Li W, Zhong Z, Chai Y, Yang L, Zheng H, Wang W, Fu H, He M, Huang X. Molecular characterization and new genotypes of *Enterocytozoon bieneusi* in pet chipmunks (*Eutamias asiaticus*) in Sichuan province, China. BMC Microbiol. 2018;18(1):37.

32. Li W, Li Y, Song M, Lu Y, Yang J, Tao W, Jiang Y, Wan Q, Zhang S, Xiao L. Prevalence and genetic characteristics of *Cryptosporidium*, *Enterocytozoon bieneusi* and *Giardia duodenalis* in cats and dogs in Heilongjiang province, China. Vet Parasitol. 2015;208(3):125-34.

33. Santín M, Calero‐Bernal R, Carmena D, Mateo M, Balseiro A, Barral M, Lima Barbero JF, Habela MÁ. Molecular characterization of *Enterocytozoon bieneusi* in wild carnivores in Spain. J Eukaryot Microbiol. 2018;65(4):468-74.

34. Ye J, Yan J, Xu J, Ma K, Yang X. Zoonotic *Enterocytozoon bieneusi* in raw wastewater in Zhengzhou, China. Folia Parasitol. 2017;64:1.

35. Feng Y, Li N, Dearen T, Lobo ML, Matos O, Cama V, Xiao L. Development of a multilocus sequence typing tool for high-resolution genotyping of *Enterocytozoon bieneusi*. Appl Environ Microbiol. 2011;77(14):4822-8.

36. Mathis A, Breitenmoser AC, Deplazes P. Detection of new *Enterocytozoon* genotypes in faecal samples of farm dogs and a cat. Parasite. 1999;6(2):189-93.

37. Piekarska J, Kicia M, Wesołowska M, Kopacz Ż, Gorczykowski M, Szczepankiewicz B, Kvac M, Sak B. Zoonotic microsporidia in dogs and cats in Poland. Vet Parasitol. 2017;246:108-11.
