# Supplementary material for: Enterocytozoon bieneusi genotypes in cats and dogs in Victoria, Australia
Source: BMC Microbiol. 2019 Aug 8;19:183. doi: 10.1186/s12866-019-1563-y (PMC6686557; doi:10.1186/s12866-019-1563-y)
Supplement: Supplementary file 2 — Table S2. Genotypes D (synonyms: CEbC, MJ10-12, NCF7, Peru9, PigEBITS9, PtEb VI, SHW1and WL8) and genotype PtEb IX (synonyms: eb52 and EntcanA) of Enterocytozoon bieneusi recorded in different host species, food and water samples fromprevious publications. (DOCX 284 kb) [file 12866_2019_1563_MOESM2_ESM.docx]

**Additional file 2: Table S2**. Genotypes D (synonyms: CEbC, MJ10-12, NCF7, Peru9, PigEBITS9, PtEb VI, SHW1and WL8) and genotype PtEb IX (synonyms: eb52 and EntcanA) of *Enterocytozoon bieneusi* recorded in different host species, food and water samples from previous publications

| Geno-type | GenBank ID | Origin | Latin name | Country | Refer-ence |
| --- | --- | --- | --- | --- | --- |
| D | KT943972 | Chicken | *Gallus gallus domesticus* | Brazil | [1] |
| D | AF101200 | Common crane | *Grus grus* | China | [2] |
| D | AF101200 | Exotic bird | NA | Iran | [3] |
| D | DQ793213 | Gyr–Peregrine falcon | *Falco rusticolus X Falco cherrug* | Abu Dubai | [4] |
| D | DQ793213 | Gyrfalcon | *Falco rusticolus* | Abu Dubai | [4] |
| D | DQ793213 | Lanner falcon | *Falco biarmicus* | Abu Dubai | [4] |
| D | DQ793213 | Peregrine falcon | *Falco peregrinus* | Abu Dubai | [4] |
| D | JF776168 | Pigeon | NA | Iran | [5] |
| D | AF101200 | Red-crowned crane *Grus japonensis* | | China | [2] |
| D | KT267295 | Red-headed lovebird | *Agapornis pullarius* | China | [6] |
| D | KX375799 | Rook | *Corvus frugilegus* | Poland | [7] |
| D | KY012355 | Swan goose | *Anser cygnoides* | Brazil | [8] |
| D | KC860906 | Alpaca | *Vicugna pacos* | Peru | [9] |
| D | KC860936 | Alpaca | *Vicugna pacos* | Peru | [9] |
| D | KT750166 | Arctic fox | *Vulpes lagopus* | China | [10] |
| D | AF101200 | Arctic fox | *Vulpes lagopus* | China | [11] |
| D | KX423961 | Asian golden cat | *Catopuma temminckii*China | | [12] |
| D | AF101200 | Asiatic black bear | *Ursus thibetanus* | China | [13] |
| D | KR062135 | Bank vole | *Myodes glareolus* | Poland | [14] |
| D | KJ728787 | Bornean orangutan *Pongo pygmaeus* | | China | [15] |
| D | AF101200 | Bornean orangutan *Pongo pygmaeus* | | China | [16] |
| D | AF101200 | Bornean orangutan *Pongo pygmaeus* | | Indonesia | [17] |
| D | AF101200 | Brown rat | *Rattus norvegicus* | China | [18] |
| D | AF023245 | Brown rat | *Rattus norvegicus* | China | [19] |
| D | AF101200 | Cat | *Felis catus* | Thailand | [20] |
| D | KJ668727 | Cat | *Felis catus* | China | [21] |
| D | AF101200 | Cat | *Felis catus* | China | [22] |
| D | KF305583 | Cat | *Felis catus* | China | [23] |
| D | KF383393 | Cat | *Felis catus* | Czech Republic | [24] |
| D | KF675191 | Cattle | *Bos taurus* | Argentina | [25] |
| D | JQ923453 | Cattle | *Bos taurus* | South Africa [26] | |
| D | AF101200 | Cattle | *Bos taurus* | China | [27] |
| D | AF101200 | Cattle | *Bos taurus* | China | [28] |
| D | KT984496 | Cattle | *Bos taurus* | Brazil | [29] |
| D | AF101200 | Cattle | *Bos taurus* | Iran | [30] |
| D | KU531574 | Cattle | *Bos taurus* | China | [31] |
| D | MF410396 | Common chipmunk *Eutamias asiaticus* | | China | [32] |
| D | KJ668727 | Dog | *Canis familiaris* | China | [21] |
| D | AF101200 | Dog | *Canis familiaris* | China | [22] |
| D | KF305583 | Dog | *Canis familiaris* | China | [23] |
| D | AF101200 | Dog | *Canis familiaris* | Poland | [33] |
| D | KU557672 | Donkey | *Equus africanus asinus* | China | [34] |
| D | KT325924 | European rabbit | *Oryctolagus cuniculus* | China | [35] |
| D | AF023245 | Fox | NA | Spain | [36] |
| D | AF101200 | Giant panda | *Ailuropoda melanoleuca* | China | [37] |
| D | KY950534 | Giant panda | *Ailuropoda melanoleuca* | China | [38] |
| D | AF101200 | Goat | *Capra aegagrus hircus* | China | [39] |
| D | KP262379 | Goat | *Capra aegagrus hircus* | China | [40] |
| D | KJ728787 | Golden monkey | *Cercopithecus kandti* China | | [15] |
| D | KM591955 | Golden snub-nosed monkey | *Rhinopithecus roxellana* | China | [41] |
| D | KM591956 | Golden snub-nosed monkey | *Rhinopithecus roxellana* | China | [41] |
| D | KU852470 | Golden snub-nosed monkey | *Rhinopithecus roxellana* | China | [12] |
| D | KU604932 | Golden snub-nosed monkey | *Rhinopithecus roxellana* | China | [42] |
| D | AF101200 | Golden snub-nosed monkey | *Rhinopithecus roxellana* | China | [16] |
| D | AF101200 | Golden takin | *Budorcas taxicolor bedfordi* | China | [43] |
| D | KJ728787 | Green monkey | *Chlorocebus sabaeus* China | | [15] |
| D | AF101200 | Green monkey | *Chlorocebus sabaeus* China | | [16] |
| D | KJ728787 | Hamadryas baboon *Papio hamadryas* | | China | [15] |
| D | KM591953 | Hamadryas baboon *Papio hamadryas* | | China | [41] |
| D | KM591954 | Hamadryas baboon *Papio hamadryas* | | China | [41] |
| D | AF101200 | Hamadryas baboon *Papio hamadryas* | | China | [16] |
| D | KT267281 | Hippopotamus | *Hippopotamus amphibious* | China | [6] |
| D | AF101200 | Horse | *Equus ferus caballus* | Czech Republic | [44] |
| D | GQ406055 | Horse | *Equus ferus caballus* Colombia | | [45] |
| D | KJ941130 | Horse | *Equus ferus caballus* Algeria | | [46] |
| D | KU194595 | Horse | *Equus ferus caballus* China | | [47] |
| D | KX276705 | Horse | *Equus ferus caballus* China | | [48] |
| D | AF101200 | House mouse | *Mus musculus domesticus* | Czech–Germany | [49] |
| D | AF101200 | Human | *Homo sapiens* | Nigeria | [50] |
| D | AF101200 | Human | *Homo sapiens* | Malawi | [51] |
| D | AF101200 | Human | *Homo sapiens* | Netherlands | [51] |
| D | AF101200 | Human | *Homo sapiens* | Cameroon | [52] |
| D | AF101200 | Human | *Homo sapiens* | Gabon | [52] |
| D | AF101200 | Human | *Homo sapiens* | Vietnam | [53] |
| D | AF101200 | Human | *Homo sapiens* | Portugal | [54] |
| D | JQ923453 | Human | *Homo sapiens* | India | [55] |
| D | AF101200 | Human | *Homo sapiens* | Thailand | [56] |
| D | JQ029731 | Human | *Homo sapiens* | China | [57] |
| D | JX994272 | Human | *Homo sapiens* | China | [58] |
| D | JF927954 | Human | *Homo sapiens* | Brazil | [59] |
| D | AF101200 | Human | *Homo sapiens* | Iran | [60] |
| D | AF101200 | Human | *Homo sapiens* | England | [61] |
| D | AF101200 | Human | *Homo sapiens* | Nigeria | [62] |
| D | AF101200 | Human | *Homo sapiens* | Thailand | [63] |
| D | AF023245 | Human | *Homo sapiens* | Russia | [64] |
| D | AF101200 | Human | *Homo sapiens* | Russia | [64] |
| D | DQ793213 | Human | *Homo sapiens* | Russia | [64] |
| D | AF101200 | Human | *Homo sapiens* | Czech Republic | [65] |
| D | AF101200 | Human | *Homo sapiens* | Tunisia | [66] |
| D | AF101200 | Human | *Homo sapiens* | Thailand | [67] |
| D | KF261748 | Human | *Homo sapiens* | Uganda | [68] |
| D | KF261751 | Human | *Homo sapiens* | Uganda | [68] |
| D | KF261779 | Human | *Homo sapiens* | Uganda | [68] |
| D | KF261814 | Human | *Homo sapiens* | Uganda | [68] |
| D | KF261858 | Human | *Homo sapiens* | Uganda | [68] |
| D | KF261889 | Human | *Homo sapiens* | Uganda | [68] |
| D | KF261906 | Human | *Homo sapiens* | Uganda | [68] |
| D | KF261907 | Human | *Homo sapiens* | Uganda | [68] |
| D | KF261955 | Human | *Homo sapiens* | Uganda | [68] |
| D | KF261957 | Human | *Homo sapiens* | Uganda | [68] |
| D | KF261980 | Human | *Homo sapiens* | Uganda | [68] |
| D | JX683802 | Human | *Homo sapiens* | Nigeria | [69] |
| D | KJ700425 | Human | *Homo sapiens* | Iran | [70] |
| D | KJ700427 | Human | *Homo sapiens* | Iran | [70] |
| D | KJ700429 | Human | *Homo sapiens* | Iran | [70] |
| D | KJ700430 | Human | *Homo sapiens* | Iran | [70] |
| D | KJ700434 | Human | *Homo sapiens* | Iran | [70] |
| D | AF101200 | Human | *Homo sapiens* | Iran | [71] |
| D | AF101200 | Human | *Homo sapiens* | Poland | [72] |
| D | JQ029731 | Human | *Homo sapiens* | China | [73] |
| D | AF101200 | Human | *Homo sapiens* | Thailand | [74] |
| D | KY066465 | Human | *Homo sapiens* | China | [75] |
| D | AF101200 | Human | *Homo sapiens* | Thailand | [76] |
| D | AF101200 | Human | *Homo sapiens* | France | [77] |
| D | AF023245 | Human | *Homo sapiens* | Poland | [78] |
| D | JQ923453 | Human | *Homo sapiens* | Nigeria | [79] |
| D | AF101200 | Human | *Homo sapiens* | Congo | [80] |
| D | KJ728787 | Hussar monkey | *Erythrocebus patas* | China | [15] |
| D | AF101200 | Hussar monkey | *Erythrocebus patas* | China | [16] |
| D | KJ728787 | King colobus | *Colobus polykomos* | China | [15] |
| D | AF101200 | King colobus | *Colobus polykomos* | China | [16] |
| D | KT267298 | Lion | *Panthera leo* | China | [6] |
| D | KU852469 | Lion | *Panthera leo* | China | [12] |
| D | AF101200 | Long-tailed chinchilla | *Chinchilla lanigera* | China | [81] |
| D | KF305583 | Long-tailed macaque | *Macaca fascicularis* | China | [82] |
| D | DQ683755 | Long-tailed macaque | *Macaca fascicularis* | China | [83] |
| D | MF476879 | Long-tailed macaque | *Macaca fascicularis* | China | [84] |
| D | AF101200 | Long-tailed macaque | *Macaca fascicularis* | China | [16] |
| D | AF101200 | Mouse | *Mus musculus musculus* | Czech–Germany | [49] |
| D | AF101200 | North American river otter | *Lontra canadensis* | USA | [85] |
| D | KJ728787 | Northern white-cheeked gibbon | *Nomascus leucogenys* | China | [15] |
| D | KX423961 | Northern white-cheeked gibbon | *Nomascus leucogenys* | China | [12] |
| D | KX905211 | Northern white-cheeked gibbon | *Nomascus leucogenys* | China | [86] |
| D | AF101200 | Northern white-cheeked gibbon | *Nomascus leucogenys* | China | [16] |
| D | DQ683751 | Olive baboon | *Papio anubis* | Kenya | [87] |
| D | KJ728787 | Olive baboon | *Papio anubis* | China | [15] |
| D | KU852471 | Olive baboon | *Papio anubis* | China | [12] |
| D | AF101200 | Olive baboon | *Papio anubis* | China | [16] |
| D | AB470282 | Pig | *Sus scrofa domesticus*Japan | | [88] |
| D | AF101200 | Pig | *Sus scrofa domesticus*China | | [89] |
| D | JF776168 | Pig | *Sus scrofa domesticus*China | | [90] |
| D | AF101200 | Pig | *Sus scrofa domesticus*USA | | [91] |
| D | AF101200 | Pig | *Sus scrofa domesticus* | Czech Republic | [92] |
| D | AF101200 | Pig | *Sus scrofa domesticus*Thailand | | [76] |
| D | MG183822 | Pig | *Sus scrofa domesticus*China | | [93] |
| D | KY495656 | Pig | *Sus scrofa domesticus*China | | [94] |
| D | AF023245 | Rabbit | NA | Spain | [36] |
| D | KU852472 | Raccoon | *Procyon lotor* | China | [12] |
| D | AF101200 | Raccoon dog | *Nyctereutes procyonoides* | China | [95] |
| D | KU847361 | Raccoon dog | *Nyctereutes procyonoides* | China | [96] |
| D | AF101200 | Raccoon dog | *Nyctereutes procyonoides* | China | [11] |
| D | AF101200 | Red fox | *Vulpes vulpes* | China | [95] |
| D | AF101200 | Red-bellied tree squirrel | *Callosciurus erythraeus* | China | [97] |
| D | AB470282 | Rex rabbit | NA | China | [98] |
| D | KF305583 | Rhesus macaque | *Macaca mulatta* | China | [82] |
| D | AF023245 | Rhesus macaque | *Macaca mulatta* | England | [99] |
| D | KJ728787 | Rhesus macaque | *Macaca mulatta* | China | [15] |
| D | KM591957 | Rhesus macaque | *Macaca mulatta* | China | [41] |
| D | KM591958 | Rhesus macaque | *Macaca mulatta* | China | [41] |
| D | KM591959 | Rhesus macaque | *Macaca mulatta* | China | [41] |
| D | KM591960 | Rhesus macaque | *Macaca mulatta* | China | [41] |
| D | KM591961 | Rhesus macaque | *Macaca mulatta* | China | [41] |
| D | KM591962 | Rhesus macaque | *Macaca mulatta* | China | [41] |
| D | KX905208 | Rhesus macaque | *Macaca mulatta* | China | [86] |
| D | KX905209 | Rhesus macaque | *Macaca mulatta* | China | [86] |
| D | KX905210 | Rhesus macaque | *Macaca mulatta* | China | [86] |
| D | AF101200 | Rhesus macaque | *Macaca mulatta* | China | [16] |
| D | MF693831 | Sambar deer | *Rusa unicolor* | Australia | [100] |
| D | AF101200 | Sheep | *Ovis aries* | China | [39] |
| D | KT267294 | Siberian tiger | *Panthera tigris altaica* | China | [6] |
| D | KX383624 | Sika deer | *Cervus nippon* | China | [101] |
| D | KR062136 | Striped field mouse *Apodemus agrarius* | | Poland | [14] |
| D | KR062137 | Striped field mouse *Apodemus agrarius* | | Poland | [14] |
| D | AF101200 | Sumatran orangutan*Pongo abelii* | | Indonesia | [17] |
| D | KJ469969 | Western lowland gorilla | *Gorilla gorilla gorilla* | Rwanda | [102] |
| D | KF305583 | White-headed langur | *Trachypithecus poliocephalus* | China | [82] |
| D | KF383389 | Wild boar | *Sus scrofa* | Austria | [103] |
| D | KF383390 | Wild boar | *Sus scrofa* | Czech Republic | [103] |
| D | KF383391 | Wild boar | *Sus scrofa* | Czech Republic | [103] |
| D | KF383392 | Wild boar | *Sus scrofa* | Czech Republic | [103] |
| D | KF383393 | Wild boar | *Sus scrofa* | Czech Republic | [103] |
| D | KF383394 | Wild boar | *Sus scrofa* | Slovak Republic | [103] |
| D | KF383395 | Wild boar | *Sus scrofa* | Czech Republic | [103] |
| D | KX670582 | Wild boar | *Sus scrofa* | China | [104] |
| D | KR062138 | Yellow necked mouse | *Apodemus flavicollis* | Poland | [14] |
| D | KR062139 | Yellow necked mouse | *Apodemus flavicollis* | Poland | [14] |
| CEbC | EF139197 | Cattle | *Bos taurus* | Korea | [105] |
| CEbC | EF139197 | Cattle | *Bos taurus* | Korea | [106] |
| MJ10 | MF522191 | Asiatic black bear | *Ursus thibetanus* | China | [13] |
| MJ11 | MF522192 | Asiatic black bear | *Ursus thibetanus* | China | [13] |
| MJ12 | MF522193 | Asiatic black bear | *Ursus thibetanus* | China | [13] |
| NCF7 | KT750165 | Arctic fox | *Vulpes lagopus* | China | [10] |
| Peru9 | AY371284 | Human | *Homo sapiens* | Peru | [107] |
| Peru9 | AY371284 | Human | *Homo sapiens* | Peru | [108] |
| Peru9 | AY371284 | Human | *Homo sapiens* | Peru | [109] |
| Peru9 | AY371284 | Human | *Homo sapiens* | Russia | [64] |
| Peru9 | AY371284 | Pig | *Sus scrofa domesticus* | Czech Republic | [92] |
| PigEBITS9 | AF348477 | Pig | *Sus scrofa domesticus*USA | | [91] |
| PtEb VI | DQ885582 | Dog | *Canis familiaris* | Portugal | [110] |
| WL8 | AY237216 | Human | *Homo sapiens* | Russia | [64] |
| WL8 | AY237216 | Muskrat | *Ondatra zibethicus* | USA | [111] |
| WL8 | AY237216 | North American beaver | *Castor canadensis* | USA | [111] |
| WL8 | AY237216 | Raccoon | *Procyon lotor* | USA | [111] |
| WL8 | AY237216 | Red fox | *Vulpes vulpes* | USA | [111] |
| D | MG491315 | Food | NA | Iran | [112] |
| D | MG491319 | Food | NA | Iran | [112] |
| D | MG491325 | Food | NA | Iran | [112] |
| D | AF101200 | Water | NA | China | [113] |
| D | AF101200 | Water | NA | China | [114] |
| D | AF101200 | Water | NA | China | [115] |
| D | AF101200 | Water | NA | China | [116] |
| D | MG491314 | Water | NA | Iran | [112] |
| D | MG491320 | Water | NA | Iran | [112] |
| D | MG491321 | Water | NA | Iran | [112] |
| D | MG491322 | Water | NA | Iran | [112] |
| D | MG491323 | Water | NA | Iran | [112] |
| D | MG491324 | Water | NA | Iran | [112] |
| D | AY371284 | Water | NA | China | [117] |
| D | AF101200 | Water | NA | Tunisia | [118] |
| D | AF023245 | Water | NA | Spain | [119] |
| SHW1 | KX190062 | Water | NA | China | [115] |
| PtEb IX | KJ668719 | Cat | *Felis catus* | China | [21] |
| PtEb IX | DQ885585 | Cat | *Felis catus* | Poland | [33] |
| PtEb IX | DQ885585 | Dog | *Canis familiaris* | Portugal | [110] |
| PtEb IX | DQ885585 | Dog | *Canis familiaris* | USA | [59] |
| PtEb IX | KX869922 | Dog | *Canis familiaris* | China | [120] |
| PtEb IX | AB359946 | Dog | *Canis familiaris* | Japan | [121] |
| PtEb IX | EU650273 | Dog | *Canis familiaris* | Colombia | [122] |
| PtEb IX | KJ668719 | Dog | *Canis familiaris* | China | [21] |
| PtEb IX | DQ885585 | Dog | *Canis familiaris* | China | [22] |
| PtEb IX | DQ885585 | Dog | *Canis familiaris* | China | [23] |
| PtEb IX | DQ885585 | Dog | *Canis familiaris* | Poland | [33] |
| PtEb IX | MG458712 | European badger | *Meles meles* | Spain | [123] |
| PtEb IX | DQ885585 | Water | NA | China | [113] |
| PtEb IX | DQ885585 | Water | NA | China | [116] |
| PtEb IX | DQ885585 | Water | NA | China | [117] |
| eb52 | AF059610 | Cat | *Felis catus* | Poland | [33] |
| EntcanA | AF059610 | Dog | *Canis familiaris* | Switzerland | [124] |

**References**

1. da Cunha MJR, Cury MC, Santín M. Widespread presence of human-pathogenic *Enterocytozoon bieneusi* genotypes in chickens. Vet Parasitol. 2016;217:108-12.

2. Zhao W, Yu S, Yang Z, Zhang Y, Zhang L, Wang R, Zhang W, Yang F, Liu A. Genotyping of *Enterocytozoon bieneusi* (Microsporidia) isolated from various birds in China. Infect Genet Evol. 2016;40:151-4.

3. Tavalla M, Mardani-Kateki M, Abdizadeh R, Soltani S, Saki J. Molecular diagnosis of potentially human pathogenic *Enterocytozoon bieneusi* and *Encephalitozoon* species in exotic birds in southwestern Iran. J Infect Public Health. 2017;11(2):192-6.

4. Müller MG, Kinne J, Schuster RK, Walochnik J. Outbreak of microsporidiosis caused by *Enterocytozoon bieneusi* in falcons. Vet Parasitol. 2008;152(1):67-78.

5. Pirestani M, Sadraei J, Forouzandeh M. Molecular characterization and genotyping of human related microsporidia in free-ranging and captive pigeons of Tehran, Iran. Infect Genet Evol. 2013;20:495-9.

6. Li J, Qi M, Chang Y, Wang R, Li T, Dong H, Zhang L. Molecular characterization of *Cryptosporidium* spp., *Giardia duodenalis*, and *Enterocytozoon bieneusi* in captive wildlife at Zhengzhou Zoo, China. J Eukaryot Microbiol. 2015;62(6):833-9.

7. Perec-Matysiak A, Wesołowska M, Leśniańska K, Buńkowska-Gawlik K, Hildebrand J, Kicia M. Survey for zoonotic microsporidian pathogens in wild living urban rooks (*Corvus frugilegus*). J Eukaryot Microbiol. 2017;64(5):721-4.

8. da Cunha MJR, Cury MC, Santín M. Molecular identification of *Enterocytozoon bieneusi*, *Cryptosporidium*, and *Giardia* in Brazilian captive birds. Parasitol Res. 2017;116(2):487-93.

9. Gómez-Puerta LA. Caracterización molecular de genotipos de *Enterocytozoon bieneusi* y ensamblajes de *Giardia duodenalis* aislados de heces de crías de alpaca (*Vicugna pacos*). Master Thesis. Universidad Nacional Mayor de San Marcos, Faculty of Veterinary Medicine, Lima, Peru, pp. 120; 2013.

10. Zhang XX, Cong W, Lou ZL, Ma JG, Zheng WB, Yao QX, Zhao Q, Zhu XQ. Prevalence, risk factors and multilocus genotyping of *Enterocytozoon bieneusi* in farmed foxes (*Vulpes lagopus*), northern China. Parasit Vectors. 2016;9(1):72.

11. Zhao W, Zhang W, Yang Z, Liu A, Zhang L, Yang F, Wang R, Ling H. Genotyping of *Enterocytozoon bieneusi* in farmed blue foxes (*Alopex lagopus*) and raccoon dogs (*Nyctereutes procyonoides*) in China. PLoS One. 2015;10(11):e0142611.

12. Li W, Deng L, Yu X, Zhong Z, Wang Q, Liu X, Niu L, Xie N, Deng J, Lei S, et al. Multilocus genotypes and broad host-range of *Enterocytozoon bieneusi* in captive wildlife at zoological gardens in China. Parasit Vectors. 2016;9(1):395.

13. Wu J, Han JQ, Shi LQ, Zou Y, Li Z, Yang JF, Huang CQ, Zou FC. Prevalence, genotypes, and risk factors of *Enterocytozoon bieneusi* in Asiatic black bear (*Ursus thibetanus*) in Yunnan province, southwestern China. Parasitol Res. 2018;117(4):1139-45.

14. Perec-Matysiak A, Buńkowska-Gawlik K, Kváč M, Sak B, Hildebrand J, Leśniańska K. Diversity of *Enterocytozoon bieneusi* genotypes among small rodents in southwestern Poland. Vet Parasitol. 2015;214(3):242-6.

15. Karim MR, Dong H, Li T, Yu F, Li D, Zhang L, Li J, Wang R, Li S, Li X, et al. Predomination and new genotypes of *Enterocytozoon bieneusi* in captive nonhuman primates in zoos in China: high genetic diversity and zoonotic significance. PLoS One. 2015;10(2):e0117991.

16. Li JQ, Dong HJ, Wang RJ, Yu FC, Wu YY, Chang YK, Wang CR, Qi M, Zhang LX. An investigation of parasitic infections and review of molecular characterization of the intestinal protozoa in nonhuman primates in China from 2009 to 2015. Int J Parasitol Parasit Wildl. 2017;6(1):8-15.

17. Mynářová A, Foitová I, Kváč M, Květoňová D, Rost M, Morrogh-Bernard H, Nurcahyo W, Nguyen C, Supriyadi S, Sak B. Prevalence of *Cryptosporidium* spp., *Enterocytozoon bieneusi*, *Encephalitozoon* spp. and *Giardia intestinalis* in wild, semi-wild and captive orangutans (*Pongo abelii* and *Pongo pygmaeus*) on Sumatra and Borneo, Indonesia. PLoS One. 2016;11(3):e0152771.

18. Zhao W, Wang J, Ren G, Yang Z, Yang F, Zhang W, Xu Y, Liu A, Ling H. Molecular characterizations of *Cryptosporidium* spp. and *Enterocytozoon bieneusi* in brown rats (*Rattus norvegicus*) from Heilongjiang province, China. Parasit Vectors. 2018;11(1):313.

19. Yu F, Qi M, Zhao Z, Lv C, Wang Y, Wang R, Zhang L. The potential role of synanthropic rodents and flies in the transmission of *Enterocytozoon bieneusi* on a dairy cattle farm in China. J Eukaryot Microbiol. 2018; <https://doi.org/10.1111/jeu.12687>. Accessed 6 September 2018.

20. Mori H, Mahittikorn A, Thammasonthijarern N, Chaisiri K, Rojekittikhun W, Sukthana Y. Presence of zoonotic *Enterocytozoon bieneusi* in cats in a temple in central Thailand. Vet Parasitol. 2013;197(3):696-701.

21. Karim MR, Dong H, Yu F, Jian F, Zhang L, Wang R, Zhang S, Rume FI, Ning C, Xiao L. Genetic diversity in *Enterocytozoon bieneusi* isolates from dogs and cats in China: host specificity and public health implications. J Clin Microbiol. 2014;52(9):3297-302.

22. Li W, Li Y, Song M, Lu Y, Yang J, Tao W, Jiang Y, Wan Q, Zhang S, Xiao L. Prevalence and genetic characteristics of *Cryptosporidium*, *Enterocytozoon bieneusi* and *Giardia duodenalis* in cats and dogs in Heilongjiang province, China. Vet Parasitol. 2015;208(3):125-34.

23. Xu H, Jin Y, Wu W, Li P, Wang L, Li N, Feng Y, Xiao L. Genotypes of *Cryptosporidium* spp., *Enterocytozoon bieneusi* and *Giardia duodenalis* in dogs and cats in Shanghai, China. Parasit Vectors. 2016;9(1):121.

24. Kváč M, Hofmannová L, Ortega Y, Holubová N, Horčičková M, Kicia M, Hlásková L, Květoňová D, Sak B, McEvoy J. Stray cats are more frequently infected with zoonotic protists than pet cats. Folia Parasitol. 2017;64:034.

25. Del Coco VF, Córdobaa MA, Bilbao G, de Almeida Castro P, Basualdo JA, Santín M. First report of *Enterocytozoon bieneusi* from dairy cattle in Argentina. Vet Parasitol. 2014;199(1):112-5.

26. Abu Samra N, Thompson PN, Jori F, Zhang H, Xiao L. *Enterocytozoon bieneusi* at the wildlife/livestock interface of the Kruger National Park, South Africa. Vet Parasitol. 2012;190(3):587-90.

27. Zhao W, Zhang W, Yang F, Zhang L, Wang R, Cao J, Shen Y, Liu A. *Enterocytozoon bieneusi* in dairy cattle in the northeast of China: genetic diversity of ITS Gene and evaluation of zoonotic transmission potential. J Eukaryot Microbiol. 2015;62(4):553-60.

28. Li J, Luo N, Wang C, Qi M, Cao J, Cui Z, Huang J, Wang R, Zhang L. Occurrence, molecular characterization and predominant genotypes of *Enterocytozoon bieneusi* in dairy cattle in Henan and Ningxia, China. Parasit Vectors. 2016;9:142.

29. da Silva Fiuza VR, Lopes CW, de Oliveira FC, Fayer R, Santín M. New findings of *Enterocytozoon bieneusi* in beef and dairy cattle in Brazil. Vet Parasitol. 2016;216:46-51.

30. Kord-Sarkachi E, Tavalla M, Beiromvand M. Molecular diagnosis of microsporidia strains in slaughtered cows of southwest of Iran. J Parasit Dis. 2017;42(1):81-6.

31. Qi M, Jing B, Jian FC, Wang RJ, Zhang SM, Wang HY, Ning CS, Zhang LX. Dominance of *Enterocytozoon bieneusi* genotype J in dairy calves in Xinjiang, northwest China. Parasitol Int. 2017;66(1):960-3.

32. Deng L, Li W, Zhong Z, Chai Y, Yang L, Zheng H, Wang W, Fu H, He M, Huang X. Molecular characterization and new genotypes of *Enterocytozoon bieneusi* in pet chipmunks (*Eutamias asiaticus*) in Sichuan province, China. BMC Microbiol. 2018;18(1):37.

33. Piekarska J, Kicia M, Wesołowska M, Kopacz Ż, Gorczykowski M, Szczepankiewicz B, Kvac M, Sak B. Zoonotic microsporidia in dogs and cats in Poland. Vet Parasitol. 2017;246:108-11.

34. Yue DM, Ma JG, Li FC, Hou JL, Zheng WB, Zhao Q, Zhang XX, Zhu XQ. Occurrence of *Enterocytozoon bieneusi* in donkeys (*Equus asinus*) in China: a public health concern. Front Microbiol. 2017;8:565.

35. Zhang XX, Jiang J, Cai YN, Wang CF, Xu P, Yang GL, Zhao Q. Molecular characterization of *Enterocytozoon bieneusi* in domestic rabbits (*Oryctolagus cuniculus*) in northeastern China. Korean J Parasitol. 2016;54(1):81-5.

36. Galván-Díaz AL, Magnet A, Fenoy S, Henriques-Gil N, Haro M, Gordo FP, Miró G, del Águila C, Izquierdo F. Microsporidia detection and genotyping study of human pathogenic *E. bieneusi* in animals from Spain. PLoS One. 2014;9(3):e92289.

37. Li W, Song Y, Zhong Z, Huang X, Wang C, Li C, Yang H, Liu H, Ren Z, Lan J. Population genetics of *Enterocytozoon bieneusi* in captive giant pandas of China. Parasit Vectors. 2017;10(1):499.

38. Li W, Zhong Z, Song Y, Gong C, Deng L, Cao Y, Zhou Z, Cao X, Tian Y, Li H. Human-pathogenic *Enterocytozoon bieneusi* in captive giant pandas (*Ailuropoda melanoleuca*) in China. Sci Rep. 2018;8:1-7.

39. Zhao W, Zhang W, Yang D, Zhang L, Wang R, Liu A. Prevalence of *Enterocytozoon bieneusi* and genetic diversity of ITS genotypes in sheep and goats in China. Infect Genet Evol. 2015;32:265-70.

40. Shi K, Li M, Wang X, Li J, Karim MR, Wang R, Zhang L, Jian F, Ning C. Molecular survey of *Enterocytozoon bieneusi* in sheep and goats in China. Parasit Vectors. 2016;9(1):23.

41. Du SZ, Zhao GH, Shao JF, Fang YQ, Tian GR, Zhang LX, Wang RJ, Wang HY, Qi M, Yu SK. *Cryptosporidium* spp., *Giardia intestinalis,* and *Enterocytozoon bieneusi* in captive non-human primates in Qinling mountains. Korean J Parasitol. 2015;53(4):395-402.

42. Yu F, Wu Y, Li T, Cao J, Wang J, Hu S, Zhu H, Zhang S, Wang R, Ning C, et al. High prevalence of *Enterocytozoon bieneusi* zoonotic genotype D in captive golden snub-nosed monkey (*Rhinopithecus roxellanae*) in zoos in China. BMC Vet Res. 2017;13(1):158.

43. Zhao GH, Du SZ, Wang HB, Hu XF, Deng MJ, Yu SK, Zhang LX, Zhu XQ. First report of zoonotic *Cryptosporidium* spp., *Giardia intestinalis* and *Enterocytozoon bieneusi* in golden takins (*Budorcas taxicolor bedfordi*). Infect Genet Evol. 2015;34:394-401.

44. Wagnerová P, Sak B, Květoňová D, Buňatová Z, Civišová H, Maršálek M, Kváč M. *Enterocytozoon bieneusi* and *Encephalitozoon cuniculi* in horses kept under different management systems in the Czech Republic. Vet Parasitol. 2012;190(3):573-7.

45. Santín M, Cortés Vecino JA, Fayer R. A zoonotic genotype of *Enterocytozoon bieneusi* in horses. J Parasitol. 2010;96(1):157-61.

46. Laatamna AE, Wagnerová P, Sak B, Květoňová D, Xiao L, Rost M, McEvoy J, Saadi AR, Aissi M, Kváč M. Microsporidia and *Cryptosporidium* in horses and donkeys in Algeria: detection of a novel *Cryptosporidium hominis* subtype family (Ik) in a horse. Vet Parasitol. 2015;208(3):135-42.

47. Qi M, Wang R, Wang H, Jian F, Li J, Zhao J, Dong H, Zhu H, Ning C, Zhang L. *Enterocytozoon bieneusi* genotypes in grazing horses in China and their zoonotic transmission potential. J Eukaryot Microbiol. 2016;63(5):591-7.

48. Deng L, Li W, Zhong Z, Gong C, Liu X, Huang X, Xiao L, Zhao R, Wang W, Feng F, et al. Molecular characterization and multilocus genotypes of *Enterocytozoon bieneusi* among horses in southwestern China. Parasit Vectors. 2016;9(1):561.

49. Sak B, Kváč M, Hanzlíková D, Albrecht T, Piálek J. The first report on natural *Enterocytozoon bieneusi* and *Encephalitozoon* spp. infections in wild east-European house mice (*Mus musculus musculus*) and west-European house mice (*M. m. domesticus*) in a hybrid zone across the Czech Republic-Germany border. Vet Parasitol. 2011;178(3):246-50.

50. Akinbo FO, Okaka CE, Omoregie R, Dearen T, Leon ET, Xiao L. Molecular epidemiologic characterization of *Enterocytozoon bieneusi* in HIV-infected persons in Benin city, Nigeria. Am J Trop Med Hyg. 2012;86(3):441-5.

51. ten Hove RJ, Van Lieshout L, Beadsworth MB, Perez MA, Spee K, Claas EC, Verweij JJ. Characterization of genotypes of *Enterocytozoon bieneusi* in immunosuppressed and immunocompetent patient groups. J Eukaryot Microbiol. 2009;56(4):388-93.

52. Breton J, Bart-Delabesse E, Biligui S, Carbone A, Seiller X, Okome-Nkoumou M, Nzamba C, Kombila M, Accoceberry I, Thellier M. New highly divergent rRNA sequence among biodiverse genotypes of *Enterocytozoon bieneusi* strains isolated from humans in Gabon and Cameroon. J Clin Microbiol. 2007;45(8):2580-9.

53. Espern A, Morio F, Miegeville M, Illa H, Abdoulaye M, Meyssonnier V, Adehossi E, Lejeune A, Cam PD, Besse B, et al. Molecular study of microsporidiosis due to *Enterocytozoon bieneusi* and *Encephalitozoon intestinalis* among human immunodeficiency virus-infected patients from two geographical areas: Niamey, Niger, and Hanoi, Vietnam. J Clin Microbiol. 2007;45(9):2999-3002.

54. Lobo ML, Xiao L, Antunes F, Matos O. Microsporidia as emerging pathogens and the implication for public health: a 10-year study on HIV-positive and -negative patients. Int J Parasitol. 2012;42(2):197-205.

55. Li W, Cama V, Akinbo FO, Ganguly S, Kiulia NM, Zhang X, Xiao L. Multilocus sequence typing of *Enterocytozoon bieneusi*: lack of geographic segregation and existence of genetically isolated sub-populations. Infect Genet Evol. 2013;14:111-9.

56. Leelayoova S, Subrungruang I, Suputtamongkol Y, Worapong J, Petmitr PC, Mungthin M. Identification of genotypes of *Enterocytozoon bieneusi* from stool samples from human immunodeficiency virus-infected patients in Thailand. J Clin Microbiol. 2006;44(8):3001-4.

57. Wang L, Zhang H, Zhao X, Zhang L, Zhang G, Guo M, Liu L, Feng Y, Xiao L. Zoonotic *Cryptosporidium* species and *Enterocytozoon bieneusi* genotypes in HIV-positive patients on antiretroviral therapy. J Clin Microbiol. 2013;51(2):557-63.

58. Wang L, Xiao L, Duan L, Ye J, Guo Y, Guo M, Liu L, Feng Y. Concurrent infections of *Giardia duodenalis, Enterocytozoon bieneusi*, and *Clostridium difficile* in children during a cryptosporidiosis outbreak in a pediatric hospital in China. PLoS Negl Trop Dis. 2013;. 7(9):e2437.

59. Feng Y, Li N, Dearen T, Lobo ML, Matos O, Cama V, Xiao L. Development of a multilocus sequence typing tool for high-resolution genotyping of *Enterocytozoon bieneusi*. Appl Environ Microbiol. 2011;77(14):4822-8.

60. Agholi M, Hatam GR, Motazedian MH. HIV/AIDS-associated opportunistic protozoal diarrhea. AIDS Res Hum Retrov. 2013;29(1):35-41.

61. Sadler F, Peake N, Borrow R, Rowl PL, Wilkins EG, Curry A. Genotyping of *Enterocytozoon bieneusi* in AIDS patients from the north west of England. J Infect. 2002;44(1):39-42.

62. Ayinmode AB, Ojuromi OT, Xiao L. Molecular identification of *Enterocytozoon bieneusi* isolates from Nigerian children. J Parasitol Res. 2011;129542:http://dx.doi.org/10.1155/2011/129542. Accessed 14 September 2011.

63. Saksirisampant W, Prownebon J, Saksirisampant P, Mungthin M, Siripatanapipong S, Leelayoova S. Intestinal parasitic infections: prevalences in HIV/AIDS patients in a Thai AIDS-care centre. Ann Trop Med Parasitol. 2009;103(7):573-81.

64. Sokolova OI, Demyanov AV, Bowers LC, Didier ES, Yakovlev AV, Skarlato SO, Sokolova YY. Emerging microsporidian infections in Russian HIV-infected patients. J Clin Microbiol. 2011;49(6):2102-8.

65. Kicia M, Wesolowska M, Jakuszko K, Kopacz Z, Sak B, Květonová D, Krajewska M, Kváč M. Concurrent infection of the urinary tract with *Encephalitozoon cuniculi* and *Enterocytozoon bieneusi* in a renal transplant recipient. J Clin Microbiol. 2014;52(5):1780-2.

66. Chabchoub N, Abdelmalek R, Breton J, Kanoun F, Thellier M, Bouratbine A, Aoun K. Genotype identification of *Enterocytozoon bieneusi* isolates from stool samples of HIV-infected Tunisian patients. Parasite. 2012;19(2):147-51.

67. Mori H, Mahittikorn A, Watthanakulpanich D, Komalamisra C, Sukthana Y. Zoonotic potential of *Enterocytozoon bieneusi* among children in rural communities in Thailand. Parasite. 2013;20:14.

68. Widmer G, Dilo J, Tumwine JK, Tzipori S, Akiyoshi DE. Frequent occurrence of mixed *Enterocytozoon bieneusi* infections in humans. Appl Environ Microbiol. 2013;79(17):5357-62.

69. Ayinmode AB, Zhang H, Dada-Adegbola HO, Xiao L. *Cryptosporidium hominis* subtypes and *Enterocytozoon bieneusi* genotypes in HIV-infected persons in Ibadan, Nigeria. Zoonoses Public Health. 2014;61(4):297-303.

70. Mirjalali H, Mirhendi H, Meamar AR, Mohebali M, Askari Z, Mirsamadi ES, Rezaeian M. Genotyping and molecular analysis of *Enterocytozoon bieneusi* isolated from immunocompromised patients in Iran. Infect Genet Evol. 2015;36:246-50.

71. Kazemi E, Tavalla M, Maraghi S, Yad MJ, Latifi M. Frequency of microsporidial infection in immunocompromised patients with staining and molecular methods based on internal transcribed spacer region gene in two cities of southwest Iran during 2013-2014. Asian J Pharm Res Health Care. 2016;9(1):7-16.

72. Kicia M, Wesolowska M, Kopacz Z, Jakuszko K, Sak B, Květonová D, Krajewska M, Kváč M. Prevalence and molecular characteristics of urinary and intestinal microsporidia infections in renal transplant recipients. Clin Microbiol Infect. 2016;22(5):462.

73. Wang T, Fan Y, Koehler AV, Ma G, Li T, Hu M, Gasser RB. First survey of *Cryptosporidium*, *Giardia* and *Enterocytozoon* in diarrhoeic children from Wuhan, China. Infect Genet Evol. 2017;51:127-31.

74. Sanyanusin S, Mori H, Prasertbun R, Pintong AR, Komalamisra C, Changbunjong T, Popruk S, Mahittikorn A. Molecular detection and genotyping of *Blastocystis and Enterocytozoon bieneusi* in humans and pigs in Nakhon Pathom province, Thiland. JITMM Proceedings. 2017;6:1-6.

75. Zhang W, Ren G, Zhao W, Yang Z, Shen Y, Sun Y, Liu A, Cao J. Genotyping of *Enterocytozoon bieneusi* and subtyping of *Blastocystis* in cancer patients: relationship to diarrhea and assessment of zoonotic transmission. Front Microbiol. 2017;8:1835.

76. Prasertbun R, Mori H, Pintong AR, Sanyanusin S, Popruk S, Komalamisra C, Changbunjong T, Buddhirongawatr R, Sukthana Y, Mahittikorn A. Zoonotic potential of *Enterocytozoon* genotypes in humans and pigs in Thailand. Vet Parasitol. 2017;233:73-9.

77. Greigert V, Pfaff AW, Abou-Bacar A, Candolfi E, Brunet J. Intestinal microsporidiosis in Strasbourg from 2014 to 2016: emergence of an *Enterocytozoon bieneusi* genotype of asian origin. Emerg Microbes Infect. 2018;7(1):97.

78. Kicia M, Sędzimirska M, Sak B, Kváč M, Wesołowska M, Hendrich AB, Kopacz Ż. Respiratory microsporidiosis caused by *Enterocytozoon bieneusi* in a HIV-negative hematopoietic stem cell transplant recipient. Int J Infect Dis. 2018;77:26-8.

79. Maikai BV, Umoh JU, Lawal IA, Kudi AC, Ejembi CL, Xiao L. Molecular characterizations of *Cryptosporidium*, *Giardia*, and *Enterocytozoon* in humans in Kaduna State, Nigeria. Exp Parasitol. 2012;131(4):452-6.

80. Wumba R, Longo-Mbenza B, Menotti J, Mandina M, Kintoki F, Situakibanza NH, Kakicha MK, Zanga J, Mbanzulu-Makola K, Nseka T, et al. Epidemiology, clinical, immune, and molecular profiles of microsporidiosis and cryptosporidiosis among HIV/AIDS patients. Int J Gen Med. 2012;5:603-11.

81. Qi M, Luo N, Wang H, Yu F, Wang R, Huang J, Zhang L. Zoonotic *Cryptosporidium* spp. and *Enterocytozoon bieneusi* in pet chinchillas (*Chinchilla lanigera*) in China. Parasitol Int. 2015;64(5):339-41.

82. Karim MR, Wang R, Dong H, Zhang L, Li J, Zhang S, Rume FI, Qi M, Jian F, Sun M, et al. Genetic polymorphism and zoonotic potential of *Enterocytozoon bieneusi* from nonhuman primates in China. Appl Environ Microbiol. 2014;80(6):1893-8.

83. Ye J, Xiao L, Li J, Huang W, Amer SE, Guo Y, Roellig D, Feng Y. Occurrence of human-pathogenic *Enterocytozoon bieneusi, Giardia duodenalis* and *Cryptosporidium* genotypes in laboratory macaques in Guangxi, China. Parasitol Int. 2014;63(1):133-7.

84. Yang H, Lin Y, Li Y, Song M, Lu Y, Li W. Molecular characterization of *Enterocytozoon bieneusi* isolates in laboratory macaques in north China: zoonotic concerns. Parasitol Res. 2017;116(10):2877-82.

85. Guo Y, Alderisio KA, Yang W, Cama V, Feng Y, Xiao L. Host specificity and source of *Enterocytozoon bieneusi* genotypes in a drinking source watershed. Appl Environ Microbiol. 2014;80(1):218-25.

86. Zhong Z, Li W, Deng L, Song Y, Wu K, Tian Y, Huang X, Hu Y, Fu H, Geng Y, et al. Multilocus genotyping of *Enterocytozoon bieneusi* derived from nonhuman primates in southwest China. PLoS One. 2017;12(5):e0176926.

87. Li W, Kiulia NM, Mwenda JM, Nyachieo A, Taylor MB, Zhang X, Xiao L. *Cyclospora papionis*, *Cryptosporidium hominis*, and human-pathogenic *Enterocytozoon bieneusi* in captive baboons in Kenya. J Clin Microbiol. 2011;49(12):4326-9.

88. Abe N, Kimata I. Molecular survey of *Enterocytozoon bieneusi* in a Japanese porcine population. Vector Borne Zoonotic Dis. 2010;10(4):425-7.

89. Li W, Diao R, Yang J, Xiao L, Lu Y, Li Y, Song M. High diversity of human-pathogenic *Enterocytozoon bieneusi* genotypes in swine in northeast China. Parasitol Res. 2014;113(3):1147-53.

90. Zhao W, Zhang W, Yang F, Cao J, Liu H, Yang D, Shen Y, Liu A. High prevalence of *Enterocytozoon bieneusi* in asymptomatic pigs and assessment of zoonotic risk at the genotype level. Appl Environ Microbiol. 2014;80(12):3699-707.

91. Buckholt MA, Lee JH, Tzipori S. Prevalence of *Enterocytozoon bieneusi* in swine: an 18-month survey at a slaughterhouse in Massachusetts. Appl Environ Microbiol. 2002;68(5):2595-9.

92. Sak B, Kváč M, Hanzlíková D, Cama V. First report of *Enterocytozoon bieneusi* infection on a pig farm in the Czech Republic. Vet Parasitol. 2008;153(3):220-4.

93. Wang SS, Li JQ, Li YH, Wang XW, Fan XC, Liu X, Li ZJ, Song JK, Zhang LX, Zhao GH. Novel genotypes and multilocus genotypes of *Enterocytozoon bieneusi* in pigs in northwestern China: a public health concern. Infect Genet Evol. 2018;63:89-94.

94. Zou Y, Hou JL, Li FC, Zou FC, Lin RQ, Ma JG, Zhang XX, Zhu XQ. Prevalence and genotypes of *Enterocytozoon bieneusi* in pigs in southern China. Infect Genet Evol. 2018;44:52-6.

95. Yang Y, Lin Y, Li Q, Zhang S, Tao W, Wan Q, Jiang Y, Li W. Widespread presence of human-pathogenic *Enterocytozoon bieneusi* genotype D in farmed foxes (*Vulpes vulpes*) and raccoon dogs (*Nyctereutes procyonoides*) in China: first identification and zoonotic concern. Parasitol Res. 2015;114(11):4357-8.

96. Xu C, Ma X, Zhang H, Zhang XX, Zhao JP, Ba HX, Rui D, Xing XM, Wang QK, Zhao Q. Prevalence, risk factors and molecular characterization of *Enterocytozoon bieneusi* in raccoon dogs (*Nyctereutes procyonoides*) in five provinces of northern China. Acta Trop. 2016;161:68-72.

97. Deng L, Li W, Yu X, Gong C, Liu X, Zhong Z, Xie N, Lei S, Yu J, Fu H, et al. First report of the human-pathogenic *Enterocytozoon bieneusi* from red-bellied tree squirrels (*Callosciurus erythraeus*) in Sichuan, China. PLoS One. 2016;11(9):e0163605.

98. Yang Z, Zhao W, Shen Y, Zhang W, Shi Y, Ren G, Yang D, Ling H, Yang F, Liu A, et al. Subtyping of *Cryptosporidium cuniculus* and genotyping of *Enterocytozoon bieneusi* in rabbits in two farms in Heilongjiang province, China. Parasite. 2016;23:52.

99. Chalifoux LV, Carville A, Pauley D, Thompson B, Lackner AA, Mansfield KG. *Enterocytozoon bieneusi* as a cause of proliferative serositis in simian immunodeficiency virus-infected immunodeficient macaques (*Macaca mulatta*). Arch Pathol Lab Med. 2000;124(10):1480-4.

100. Zhang Y, Koehler AV, Wang T, Haydon SR, Gasser RB. First detection and genetic characterisation of *Enterocytozoon bieneusi* in wild deer in Melbourne’s water catchments in Australia. Parasit Vectors. 2018;11(1):2.

101. Huang J, Zhang Z, Yang Y, Wang R, Zhao J, Jian F, Ning C, Zhang L. New genotypes of *Enterocytozoon bieneusi* isolated from sika deer and red deer in China. Front Microbiol. 2017;8:879.

102. Sak B, Petrželková KJ, Květoňová D, Mynářová A, Pomajbíková K, Modrý D, Cranfield MR, Mudakikwa A, Kváč M. Diversity of microsporidia, *Cryptosporidium* and *Giardia* in mountain gorillas (*Gorilla beringei beringei*) in Volcanoes National Park, Rwanda. PLoS One. 2014;9(11):e109751.

103. Němejc K, Sak B, Květoňová D, Hanzal V, Janiszewski P, Forejtek P, Rajský D, Kotková M, Ravaszová P, McEvoy J, et al. Prevalence and diversity of *Encephalitozoon* spp. and *Enterocytozoon bieneusi* in wild boars (*Sus scrofa*) in central Europe. Parasitol Res. 2014;113(2):761-7.

104. Li W, Deng L, Wu K, Huang X, Song Y, Su H, Hu Y, Fu H, Zhong Z, Peng G. Presence of zoonotic *Cryptosporidium scrofarum*, *Giardia duodenalis* assemblage A and *Enterocytozoon bieneusi* genotypes in captive Eurasian wild boars (*Sus scrofa*) in China: potential for zoonotic transmission. Parasit Vectors. 2017;10(1):10.

105. Lee JH. Prevalence and molecular characteristics of *Enterocytozoon bieneusi* in cattle in Korea. Parasitol Res. 2007;101(2):391-6.

106. Lee JH. Molecular detection of *Enterocytozoon bieneusi* and identification of a potentially human-pathogenic genotype in milk. Appl Environ Microbiol. 2008;74(5):1664-6.

107. Cama VA, Pearson J, Cabrera L, Pacheco L, Gilman R, Meyer S, Ortega Y, Xiao L. Transmission of *Enterocytozoon bieneusi* between a child and guinea pigs. J Clin Microbiol. 2007;45(8):2708-10.

108. Sulaiman IM, Bern C, Gilman R, Cama V, Kawai V, Vargas D, Ticona E, Vivar A, Xiao L. A molecular biologic study of *Enterocytozoon bieneusi* in HIV-infected patients in Lima, Peru. J Eukaryot Microbiol. 2003;50 Suppl 1:591-6.

109. Bern C, Kawai V, Vargas D, Rabke-Verani J, Williamson J, Chavez-Valdez R, Xiao L, Sulaiman I, Vivar A, Ticona E, et al. The epidemiology of intestinal microsporidiosis in patients with HIV/AIDS in Lima, Peru. J Infect Dis. 2005;191(10):1658-64.

110. Lobo ML, Xiao L, Cama V, Stevens T, Antunes F, Matos O. Genotypes of *Enterocytozoon bieneusi* in mammals in Portugal. J Eukaryot Microbiol. 2006;53 Suppl 1:61-4.

111. Sulaiman IM, Fayer R, Lal AA, Trout JM, Schaefer FW, Xiao L. Molecular characterization of microsporidia indicates that wild mammals harbor host-adapted *Enterocytozoon* spp. as well as human-pathogenic *Enterocytozoon bieneusi*. Appl Environ Microbiol. 2003;69(8):4495-501.

112. Javanmard E, Mirjalali H, Niyyati M, Jalilzadeh E, Tabaei SJS, Aghdaei HA, Nazemalhosseini-Mojarad E, Zali MR. Molecular and phylogenetic evidences of dispersion of human-infecting microsporidia to vegetable farms via irrigation with treated wastewater: one-year follow up. Int J Hyg Environ Health. 2018;221(4):642-51.

113. Hu Y, Feng Y, Huang C, Xiao L. Occurrence, source, and human infection potential of *Cryptosporidium* and *Enterocytozoon bieneusi* in drinking source water in Shanghai, China, during a pig carcass disposal incident. Environ Sci Technol. 2014;48(24):14219-27.

114. Ma J, Feng Y, Hu Y, Villegas EN, Xiao L. Human infective potential of *Cryptosporidium* spp., *Giardia duodenalis* and *Enterocytozoon bieneusi* in urban wastewater treatment plant effluents. J Water Health. 2016;14(3):411-23.

115. Huang C, Hu Y, Wang L, Wang Y, Li N, Guo Y, Feng Y, Xiao L. Environmental transport of emerging human-pathogenic *Cryptosporidium* species and subtypes through combined sewer overflow and wastewater. Appl Environ Microbiol. 2017;83(16):e00682-17.

116. Ye J, Yan J, Xu J, Ma K, Yang X. Zoonotic *Enterocytozoon bieneusi* in raw wastewater in Zhengzhou, China. Folia Parasitol. 2017;64:1.

117. Li N, Xiao L, Wang L, Zhao S, Zhao X, Duan L, Guo M, Liu L, Feng Y. Molecular surveillance of *Cryptosporidium* spp., *Giardia duodenalis*, and *Enterocytozoon bieneusi* by genotyping and subtyping parasites in wastewater. PLoS Negl Trop Dis. 2012;6(9):e1809.

118. Ayed LB, Yang W, Widmer G, Cama V, Ortega Y, Xiao L. Survey and genetic characterization of wastewater in Tunisia for *Cryptosporidium* spp., *Giardia duodenalis*, *Enterocytozoon bieneusi*, *Cyclospora cayetanensis* and *Eimeria* spp. J Water Health. 2012;10(3):431-44.

119. Galván AL, Magnet A, Izquierdo F, Fenoy S, Rueda C, Fernandez Vadillo C, Henriques-Gil N, del Águila C. Molecular characterization of human-pathogenic microsporidia and *Cyclospora cayetanensis* isolated from various water sources in Spain: a year-long longitudinal study. Appl Environ Microbiol. 2013;79(2):449-59.

120. Li WC, Qin J, Wang K, Gu YF. Genotypes of *Enterocytozoon bieneusi* in dogs and cats in eastern China. Iran J Parasit. 2018;13(3):457-65.

121. Abe N, Kimata I, Iseki M. Molecular evidence of *Enterocytozoon bieneus*i in Japan. J Vet Med Sci. 2009;71(2):217-9.

122. Santín M, Cortés Vecino JA, Fayer R. *Enterocytozoon bieneusi* genotypes in dogs in Bogota, Colombia. Am J Trop Med Hyg. 2008;79(2):215-7.

123. Santín M, Calero‐Bernal R, Carmena D, Mateo M, Balseiro A, Barral M, Lima Barbero JF, Habela MÁ. Molecular characterization of *Enterocytozoon bieneusi* in wild carnivores in Spain. J Eukaryot Microbiol. 2018;65(4):468-74.

124. Mathis A, Breitenmoser AC, Deplazes P. Detection of new *Enterocytozoon* genotypes in faecal samples of farm dogs and a cat. Parasite. 1999;6(2):189-93.
