# Supplementary material for: Enterocytozoon bieneusi genotypes in cats and dogs in Victoria, Australia
Source: BMC Microbiol. 2019 Aug 8;19:183. doi: 10.1186/s12866-019-1563-y (PMC6686557; doi:10.1186/s12866-019-1563-y)
Supplement: Supplementary file 3 — Table S3. All Enterocytozoon bieneusi genotypes, prevalence and risk factors recorded previously in cats (Felis catus) and dogs (Canis lupus familiaris) worldwide. (DOCX 75 kb) [file 12866_2019_1563_MOESM3_ESM.docx]

**Additional file 3: Table S3.** All *Enterocytozoon bieneusi* genotypes, prevalence and risk factors recorded previously in cats (*Felis catus*) and dogs (*Canis lupus familiaris*) worldwide

| Genotype | GenBank ID | Host (Source) | Prevalence of *E. bieneusi* % (*E. bieneusi* positive sample nos./total sample nos.) | Country | Reference |
| --- | --- | --- | --- | --- | --- |
| Type IV | AF242478 | Cat (P) | 1.4 (2/143) | China | [1] |
| D | AF023245 | Cat (P) | 1.4 (2/143) | China | [1] |
| D | MH161409 | Cat (H) | 3.3 (2/60) | Brazil | [2] |
| D | KF383393 | Cat (S) | 4.8 (3/63) | Czech Republic | [3] |
| L | AF267142 | Cat (NA) | 5 (3/60) | Germany | [4] |
| K | AF267141 | Cat (NA) | 5 (3/60) | Germany | [4] |
| D | MG570078 | Cat (H) | 5.6 (4/72) | Turkey | [5] |
| Type IV | MG727664 | Cat (H) | 5.6 (4/72) | Turkey | [5] |
| Type IV | KF305582 | Cat (P) | 5.6 (9/160) | China | [6] |
| D | KF305583 | Cat (P) | 5.6 (9/160) | China | [6] |
| Type IV | AF242478 | Cat (P) | 5.8 (3/52) | China | [7] |
| D | AF101200 | Cat (P) | 5.8 (3/52) | China | [7] |
| EbfelA | AF118144 | Cat (F) | 8.3 (1/12) | Switzerland | [8] |
| PtEb IX | DQ885585 | Cat (H) | 9.1 (4/44) | Poland | [9] |
| eb52 | AF059610 | Cat (H) | 9.1 (4/44) | Poland | [9] |
| PtEb IX | KJ668719 | Cat (P) | 11.5 (11/96) | China | [10] |
| CC1 | KJ668739 | Cat (P) | 11.5 (11/96) | China | [10] |
| CC2 | KJ668740 | Cat (P) | 11.5 (11/96) | China | [10] |
| CC3 | KJ668741 | Cat (P) | 11.5 (11/96) | China | [10] |
| CC4 | KJ668742 | Cat (P) | 11.5 (11/96) | China | [10] |
| D | KJ668727 | Cat (P) | 11.5 (11/96) | China | [10] |
| I | KJ668738 | Cat (P) | 11.5 (11/96) | China | [10] |
| BEB6 | KJ668737 | Cat (P) | 11.5 (11/96) | China | [10] |
| D | KF383393 | Cat (S) | 12.1 (4/33) | Slovak Republic | [3] |
| D | KF383393 | Cat (S) | 12.8 (5/39) | Poland | [3] |
| K | AB359945 | Cat (S) | 14.3 (1/7) | Japan | [11] |
| D-like | DQ836345 | Cat (S) | 17.4 (8/46) | Colombia | [12] |
| Peru10 | DQ836342 | Cat (S) | 17.4 (8/46) | Colombia | [12] |
| K | DQ836343 | Cat (S) | 17.4 (8/46) | Colombia | [12] |
| Peru5 | DQ836344 | Cat (S) | 17.4 (8/46) | Colombia | [12] |
| ETMK2 | KC509909 | Cat (S) | 31.3 (25/80) | Thailand | [13] |
| ETMK3 | KC509910 | Cat (S) | 31.3 (25/80) | Thailand | [13] |
| ETMK4 | KC509911 | Cat (S) | 31.3 (25/80) | Thailand | [13] |
| D | AF101200 | Cat (S) | 31.3 (25/80) | Thailand | [13] |
| PtEb IV | DQ885580 | Cat (P) | 100 (6/6)^a^ | Portugal | [14] |
| PtEb VIII | DQ885584 | Cat (P) | 100 (6/6)^a^ | Portugal | [14] |
| PtEb III | DQ885579 | Cat (P) | 100 (6/6)^a^ | Portugal | [14] |
| PtEb IX | AB359946 | Dog (P, S) | 2.5 (2/79) | Japan | [14] |
| PtEb IX | DQ885585 | Dog (H) | 4.9 (4/82) | Poland | [9] |
| D | AF101200 | Dog (H) | 4.9 (4/82) | Poland | [9] |
| PtEb IX | DQ885585 | Dog (H, P) | 6.0 (29/485) | China | [6] |
| D | KF305583 | Dog (H, P) | 6.0 (29/485) | China | [6] |
| PtEb IX | DQ885585 | Dog (P) | 7.2 (18/249) | China | [7] |
| NED1 | KM870515 | Dog (P) | 7.2 (18/249) | China | [7] |
| NED2 | KM870516 | Dog (P) | 7.2 (18/249) | China | [7] |
| NED3 | KM870517 | Dog (P) | 7.2 (18/249) | China | [7] |
| NED4 | KM870518 | Dog (P) | 7.2 (18/249) | China | [7] |
| EbpC | AF076042 | Dog (P) | 7.2 (18/249) | China | [7] |
| D | AF101200 | Dog (P) | 7.2 (18/249) | China | [7] |
| CHN5 | HM992513 | Dog (P) | 7.7 (2/26) | China | [15] |
| CHN6 | HM992514 | Dog (P) | 7.7 (2/26) | China | [15] |
| EntcanA | AF059610 | Dog (F) | 8.3 (3/36) | Switzerland | [8] |
| PtEb IX | KX869922 | Dog (P) | 8.6 (27/315) | China | [1] |
| CHD3 | KX869924 | Dog (P) | 8.6 (27/315) | China | [1] |
| CHD2 | KX869925 | Dog (P) | 8.6 (27/315) | China | [1] |
| CHD1 | KX869926 | Dog (P) | 8.6 (27/315) | China | [1] |
| EbpC | KX869923 | Dog (P) | 8.6 (27/315) | China | [1] |
| A | AF101197 | Dog (H) | 9.6 (7/73) | Spain | [16] |
| PtEb IX | EU650273 | Dog (S) | 15 (18/120) | Colombia | [17] |
| K | EU650272 | Dog (S) | 15 (18/120) | Colombia | [17] |
| Peru5 | EU650271 | Dog (S) | 15 (18/120) | Colombia | [17] |
| PtEb IX | KJ668719 | Dog (P, S) | 15.5 (54/348) | China | [10] |
| CM1 | KJ668720 | Dog (P, S) | 15.5 (54/348) | China | [10] |
| CD1 | KJ668728 | Dog (P, S) | 15.5 (54/348) | China | [10] |
| CD2 | KJ668729 | Dog (P, S) | 15.5 (54/348) | China | [10] |
| CD3 | KJ668730 | Dog (P, S) | 15.5 (54/348) | China | [10] |
| CD4 | KJ668731 | Dog (P, S) | 15.5 (54/348) | China | [10] |
| CD5 | KJ668732 | Dog (P, S) | 15.5 (54/348) | China | [10] |
| CD6 | KJ668733 | Dog (P, S) | 15.5 (54/348) | China | [10] |
| CD7 | KJ668734 | Dog (P, S) | 15.5 (54/348) | China | [10] |
| CD8 | KJ668735 | Dog (P, S) | 15.5 (54/348) | China | [10] |
| CD9 | KJ668736 | Dog (P, S) | 15.5 (54/348) | China | [10] |
| EbpC | KJ668723 | Dog (P, S) | 15.5 (54/348) | China | [10] |
| Type IV | KJ668722 | Dog (P, S) | 15.5 (54/348) | China | [10] |
| O | KJ668724 | Dog (P, S) | 15.5 (54/348) | China | [10] |
| D | KJ668727 | Dog (P, S) | 15.5 (54/348) | China | [10] |
| Peru8 | KJ668721 | Dog (P, S) | 15.5 (54/348) | China | [10] |
| EbpA | KJ668725 | Dog (P, S) | 15.5 (54/348) | China | [10] |
| PigEBITS5 | KJ668726 | Dog (P, S) | 15.5 (54/348) | China | [10] |
| PtEb IX | DQ885585 | Dog (NA) | 100 (1/1)^a^ | USA | [18] |
| PtEb IX | DQ885585 | Dog (P) | 100 (3/3)^a^ | Portugal | [14] |
| PtEb VII | DQ885583 | Dog (P) | 100 (3/3)^a^ | Portugal | [14] |
| PtEb VI | DQ885582 | Dog (P) | 100 (3/3)^a^ | Portugal | [14] |

^a^ = Genotyping study using previously confirmed *E. bieneusi* isolates. F = farmed animals. H = household animals. NA = not available. P = pets. S = stray animals. W = wildlife. Z = animals in a zoo. Genotypes EbpC and WL13 are synonyms; K, PtEb III and Type IV are synonyms; Peru5 and WL11 are synonyms; D, NCF7, PtEb VI and WL8 are synonyms; eb52, EntcanA and PtEb IX are synonyms.

**References**

1. Li WC, Qin J, Wang K, Gu YF. Genotypes of *Enterocytozoon bieneusi* in dogs and cats in eastern China. Iran J Parasit. 2018;13(3):457-65.

2. Prado JBF, Ramos CAdN, Fiuza VRdS, Terra VJB. Occurrence of zoonotic *Enterocytozoon bieneusi* in cats in Brazil. Rev Bras Parasitol Vet. 2019; http://dx.doi.org/10.1590/s1984-296120180096. Accessed 14 Feburary 2019.

3. Kváč M, Hofmannová L, Ortega Y, Holubová N, Horčičková M, Kicia M, Hlásková L, Květoňová D, Sak B, McEvoy J. Stray cats are more frequently infected with zoonotic protists than pet cats. Folia Parasitol. 2017;64:034.

4. Dengjel B, Zahler M, Hermanns W, Heinritzi K, Spillmann T, Thomschke A, Loscher T, Gothe R, Rinder H. Zoonotic potential of *Enterocytozoon bieneusi*. J Clin Microbiol. 2001;39(12):4495-9.

5. Pekmezci D, Pekmezci GZ, Yildirim A, Duzlu O, Inci A. Molecular detection of zoonotic microsporidia in domestic cats in Turkey: a preliminary study. Acta Parasitol. 2019; https://doi.org/10.2478/s11686-018-00003-x. Accessed 15 January 2019

6. Xu H, Jin Y, Wu W, Li P, Wang L, Li N, Feng Y, Xiao L. Genotypes of *Cryptosporidium* spp., *Enterocytozoon bieneusi* and *Giardia duodenalis* in dogs and cats in Shanghai, China. Parasit Vectors. 2016;9(1):121.

7. Li W, Li Y, Song M, Lu Y, Yang J, Tao W, Jiang Y, Wan Q, Zhang S, Xiao L. Prevalence and genetic characteristics of *Cryptosporidium*, *Enterocytozoon bieneusi* and *Giardia duodenalis* in cats and dogs in Heilongjiang province, China. Vet Parasitol. 2015;208(3):125-34.

8. Mathis A, Breitenmoser AC, Deplazes P. Detection of new *Enterocytozoon* genotypes in faecal samples of farm dogs and a cat. Parasite. 1999;6(2):189-93.

9. Piekarska J, Kicia M, Wesołowska M, Kopacz Ż, Gorczykowski M, Szczepankiewicz B, Kvac M, Sak B. Zoonotic microsporidia in dogs and cats in Poland. Vet Parasitol. 2017;246:108-11.

10. Karim MR, Dong H, Yu F, Jian F, Zhang L, Wang R, Zhang S, Rume FI, Ning C, Xiao L. Genetic diversity in *Enterocytozoon bieneusi* isolates from dogs and cats in China: host specificity and public health implications. J Clin Microbiol. 2014;52(9):3297-302.

11. Abe N, Kimata I, Iseki M. Molecular evidence of *Enterocytozoon bieneus*i in Japan. J Vet Med Sci. 2009;71(2):217-9.

12. Santín M, Trout JM, Cortés Vecino JA, Dubey JP, Fayer R. *Cryptosporidium*, *Giardia* and *Enterocytozoon bieneusi* in cats from Bogota (Colombia) and genotyping of isolates. Vet Parasitol. 2006;141(3):334-9.

13. Mori H, Mahittikorn A, Thammasonthijarern N, Chaisiri K, Rojekittikhun W, Sukthana Y. Presence of zoonotic *Enterocytozoon bieneusi* in cats in a temple in central Thailand. Vet Parasitol. 2013;197(3):696-701.

14. Lobo ML, Xiao L, Cama V, Stevens T, Antunes F, Matos O. Genotypes of *Enterocytozoon bieneusi* in mammals in Portugal. J Eukaryot Microbiol. 2006;53 Suppl 1: 61-4.

15. Zhang X, Wang Z, Su Y, Liang X, Sun X, Peng S, Lu H, Jiang N, Yin J, Xiang M, et al. Identification and genotyping of *Enterocytozoon bieneusi* in China. J Clin Microbiol. 2011;49(5):2006-8.

16. Galván-Díaz AL, Magnet A, Fenoy S, Henriques-Gil N, Haro M, Gordo FP, Miró G, del Águila C, Izquierdo F. Microsporidia detection and genotyping study of human pathogenic *E. bieneusi* in animals from Spain. PLoS One. 2014;9(3):e99289.

17. Santín M, Cortés Vecino JA, Fayer R. *Enterocytozoon bieneusi* genotypes in dogs in Bogota, Colombia. Am J Trop Med Hyg. 2008;79(2):215-7.

18. Feng Y, Li N, Dearen T, Lobo ML, Matos O, Cama V, Xiao L. Development of a multilocus sequence typing tool for high-resolution genotyping of *Enterocytozoon bieneusi*. Appl Environ Microbiol. 2011;77(14):4822-8.
